# Strategies for Incorporating Natural Therapeutic Agents into Hydrogel Dressings: Innovations in Wound Healing

**DOI:** 10.3390/polym17233105

**Published:** 2025-11-22

**Authors:** Ion Cosmin Călina, Anca Scărișoreanu, Maria Demeter, Elena Mănăilă, Gabriela Crăciun

**Affiliations:** National Institute for Laser, Plasma and Radiation Physics, 409 Atomiștilor St., 077125 Măgurele, Romania; calina.cosmin@inflpr.ro (I.C.C.); elena.manaila@inflpr.ro (E.M.); gabriela.craciun@inflpr.ro (G.C.)

**Keywords:** natural therapeutic extracts, hydrogel, wound healing, synergistic, biocompatibility

## Abstract

Effective wound management demands novel therapeutic strategies that overcome the limitations of medication by reducing inflammation, preventing infection, and accelerating tissue regeneration. The present review provides an extensive examination of natural therapeutic agents incorporated into polymeric hydrogels for wound healing purposes. Significant focus has been paid towards extraction techniques that validate the standardization, purity, and biological efficacy of natural compounds, alongside several principal incorporation strategies: direct mixing, in situ incorporation, post-loading, and nano/microencapsulation, aimed at optimizing the stability of bioactive molecules within hydrogel matrices. Representative in vitro and in vivo studies are summarized to highlight the bioactive and therapeutic effects of hybrid systems based on polymeric hydrogels. Collectively, reported evidence indicates that natural-extract-loaded hydrogels accelerate wound healing through multiple complementary mechanisms, including inflammation modulation, antimicrobial protection, moisture balance, and enhanced tissue regeneration. Furthermore, synergistic mixtures of bioactive compounds have demonstrated enhanced antimicrobial and regenerative efficacy compared to single-component formulations. Overall, bioactive hydrogels incorporating standardized or nano-encapsulated natural extracts represent a new generation of multifunctional, non-pharmaceutic wound dressings that provide excellent biocompatibility and enhanced tissue regeneration in both acute and chronic wound healing.

## 1. Introduction

The skin, as the body’s primary barrier, protects internal organs from mechanical, chemical, thermal, and most importantly pathogenic insults. When this integrity is compromised, a complex and highly coordinated biological process of wound healing is triggered to restore tissue structure and function. However, conventional wound-care therapies, which often rely on topical antibiotics, face significant limitations. Their extensive and uncontrolled use has accelerated the emergence of antibiotic-resistant strains, transforming otherwise treatable infections into chronic, non-healing wounds. Moreover, traditional dressings may cause discomfort and disrupt newly formed tissue upon replacement, while frequently failing to provide an optimal healing microenvironment [1].

Consequently, there is a pressing need to develop alternative therapeutic strategies that exert multi-targeted actions, simultaneously reducing inflammation and infection, protecting cells from oxidative stress, and accelerating tissue regeneration without exacerbating antibiotic resistance. Medicinal plants represent a valuable source of bioactive compounds capable of modulating multiple biological processes involved in wound healing. Phytochemicals such as flavonoids, alkaloids, terpenoids, and phenolic acids have been shown to attenuate inflammation, combat microbial invasion, scavenge reactive oxygen species, and stimulate collagen synthesis and fibroblast proliferation [2].

Since ancient times, natural therapeutic agents with pharmacological properties have been applied for wound treatment and infection prevention, offering a safe and sustainable alternative to antibiotic-based interventions. Numerous plant-derived extracts, *Aloe vera*, *Curcuma longa* (Turmeric), *Azadirachta indica* (Neem), *Camellia sinensis* (Green tea), *Centella asiatica*, *Calendula officinalis* (Marigold), *Matricaria chamomilla* L. (Chamomile), and *Hypericum perforatum* (St. John’s Wort), along with bee-derived products such as honey and propolis, have demonstrated advanced healing potential. An emerging and highly promising direction involves the incorporation of standardized and non-standardized natural extracts into medical devices, such as hydrogels [3], dressings, or sprayable systems, designed for topical wound applications [4]. The selection between standardized and crude extracts must be guided by the phytochemical composition, mechanism of action, and—critically—the scientific evidence, ensuring both efficacy and safety. Among the various carriers explored, hydrogels have gained considerable attention due to their unique physicochemical properties and high biocompatibility [5]. Their porous, three-dimensional structure mimics the extracellular matrix of natural tissues [6], providing an ideal scaffold for cell proliferation, migration, and nutrient exchange. Composed of 80–90% water, hydrogels maintain a moist wound environment, allow oxygen and vapor permeability, and promote angiogenesis, key events in tissue repair [7]. Furthermore, they are non-adherent, minimizing pain and secondary damage during dressing changes [8].

The use of hydrogels as delivery matrices for plant-based therapeutic agents represents a major advance in wound-care technology. By integrating the controlled-release capacity of polymeric hydrogels with the multifunctional biological activity of natural extracts, these hybrid systems provide a non-antibiotic approach to accelerate wound healing. This synergistic combination creates an optimal environment for tissue regeneration by effectively reducing inflammation and infection, mitigating oxidative stress, and stimulating angiogenesis and collagen deposition [9].

In recent years, the number of studies investigating hydrogel systems incorporating natural extracts has increased significantly, reflecting growing scientific interest in sustainable and bioactive wound dressings. *Aloe vera*, *Curcuma longa*, and *Centella asiatica* remain among the most frequently investigated extracts due to their high reproducibility and well-characterized pharmacological profiles. Despite these promising findings, challenges persist regarding the standardization of extraction procedures, the reproducibility of bioactivity, and the stability of hydrogel extract systems under physiological conditions, parameters that directly influence the biological performance and clinical applicability of these materials. The central research question of this review concerns how extraction, purification, and incorporation strategies shape the stability, bioactivity, and wound-healing performance of natural therapeutic agents embedded in hydrogel matrices. In particular, it examines how these processing steps influence physicochemical properties, release profiles, and biocompatibility, thereby outlining key design principles for advanced bioactive wound dressings.

This review aims to provide a comprehensive analysis of the methodologies and effectiveness of incorporating natural therapeutic extracts into polymeric hydrogel matrices for wound-healing applications. In addition to synthesizing current evidence, the review introduces a distinctive perspective by comparing standardized and non-standardized extracts and by outlining emerging incorporation and encapsulation technologies that enhance the stability and functional performance of natural therapeutic agents within hydrogel systems. Emphasis is placed on the critical role of extraction methods, which determine the standardization and purity of natural compounds, as well as on the incorporation strategies employed during hydrogel formulation and their subsequent biological, therapeutic, and bioactive effects, as demonstrated by in vitro and in vivo studies. By addressing existing knowledge gaps and drawing attention to several natural therapeutic agents that remain underrepresented in previous hydrogel-focused literature, this review highlights the potential of these hybrid hydrogel systems as promising, sustainable and non-pharmaceutical alternatives capable of promoting angiogenesis, collagen formation, cell proliferation, and modulation of the inflammatory response throughout the wound-healing process.

## 2. Natural Therapeutic Agents in Hydrogel Dressings for Wound Healing

### 2.1. Plant-Derived Natural Extracts

**Figure 1:**
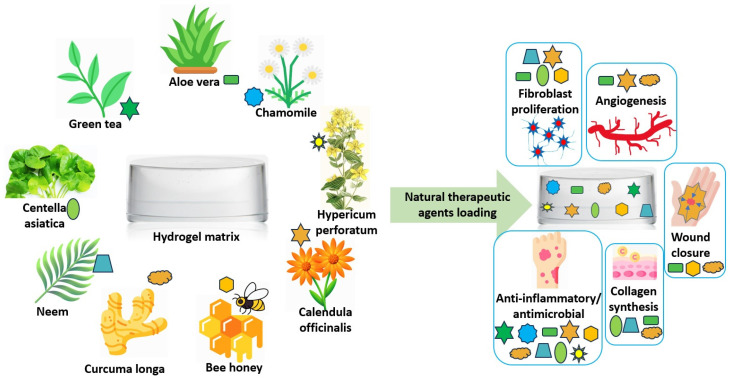
Mechanistic diagram illustrating how natural therapeutic agents are incorporated into hydrogel matrices and contribute to key pathways in the wound-healing network, including anti-inflammatory and antimicrobial activity, fibroblast proliferation, collagen synthesis, angiogenesis, and wound closure.

Therapeutic compounds derived from medicinal plants have been extensively used in the treatment of cutaneous injuries due to their diverse biological properties (Figure 1).

*Aloe vera* leaves consist of three distinct layers (green epidermis, bitter latex outer pulp, and gel-containing inner pulp), each characterized by specific phytochemical compositions. The epidermis and latex are rich in anthraquinones and phenolics, while the inner pulp contains high levels of polysaccharides such as acemannan, which collectively contribute to the plant’s therapeutic versatility [10,11]. The wound-healing potential of *Aloe vera* is primarily associated with its anti-inflammatory and enzymatic components, including C-glycosyl chromone [12], carboxypeptidase, bradykinase [13] and the polysaccharide acemannan [14]. In addition, *Aloe vera* exhibits antibacterial, antifungal, antioxidant, and immunomodulatory effects by downregulating immunosuppressive cytokines such as interleukin-10 secreted by epidermal keratinocytes. Furthermore, *Aloe vera* modulates oxidative stress through activation of the Nrf2 pathway and exerts anti-inflammatory effects via inhibition of COX-2 [15]. Preclinical studies confirm that ethanolic extracts of *Aloe vera*, rich in phytosterols and fatty acids, enhance wound closure and collagen synthesis more effectively than crude gel, particularly in thermal or traumatic injuries [16]. Commercial hydrogels incorporating *Aloe vera* (e.g., Restauder^®^) demonstrated complete wound healing within 21 days in rats, comparable to standard care, confirming the extract’s compatibility with hydrogel matrices [17]. Similarly, CS (chitosan)/*Aloe vera* composite hydrogels (50:50 ratio) significantly reduced inflammation after three days and accelerated healing within 14 days by promoting epidermal thickening and wound contraction [18].

Curcumin, demethoxycurcumin, and bisdemethoxycurcumin are bioactive curcuminoids found in *Curcuma longa* L. that have been demonstrated to have anti-inflammatory, anti-cancer, and anti-aging activities. Their anti-inflammatory effects are primarily mediated by suppression of the NF-κB signaling pathway, which leads to a reduction in the expression of pro-inflammatory cytokines [19]. Curcumin facilitates angiogenesis via the upregulation of transforming growth factor (TGF-β) and vascular endothelial growth factor (VEGF) during the wound-healing process [20]. When incorporated into 20% Pluronic F127 hydrogels, curcumin reduced the healing period to 12 days in in vivo mouse models, surpassing the performance of blank hydrogels by stimulating collagen synthesis in granulation tissue [21]. In Sprague–Dawley rats, lyophilized CS/alginate/curcumin sponges accelerated healing within 12 days and promoted ordered collagen fiber deposition [22].

Bioactive constituents of *Azadirachta indica* (Neem), including sodium nimbidate, nimbidin, terpenoids, and flavonoids, display large antiviral, antibacterial, antifungal, and anti-inflammatory activities essential for collagen deposition and neovascularization. The anti-inflammatory effects are partly mediated through suppression of TNF-α production and reduction of prostaglandin E2 levels [23]. In rat wound models, topical application of ethanolic neem extract ointment achieved complete healing within 12 days, while oral administration of neem leaf powder required 14 days, demonstrating the superior efficacy of topical delivery [24]. Similarly, guar-gum (GG)-based hydrogels loaded with neem extract enhanced tissue regeneration and achieved full wound closure within 21 days compared with untreated controls [25].

Green tea (*Camellia sinensis*) is rich in polyphenolic compounds, particularly catechins such as epigallocatechin-3-gallate (EGCG), which exhibit strong antioxidant [26], anti-inflammatory, antimicrobial [27], and angiogenic activities. These biological effects are primarily mediated by EGCG through inhibition of the NF-κB signaling pathway and activation of the Nrf2 antioxidant pathway [28]. Mice treated with EGCG-loaded PLGA membranes displayed increased vascularization and accelerated re-epithelialization compared with control membranes, confirming the compound’s pro-regenerative effects. Nevertheless, maintaining optimal EGCG concentration in hydrogel formulations is crucial, as excessive doses may exert cytotoxic effects [29].

*Centella asiatica* (CA) contains triterpene sapogenins and glycosides such as asiaticoside, madecassoside, and terminolic acid, which enhance fibroblast proliferation and stimulate type I collagen synthesis [30,31]. The plant also exhibits antibacterial, antioxidant, and anti-inflammatory properties that vary according to the extraction solvent. These biological activities are mechanistically linked to activation of the TGF-β/Smad pathway and upregulation of VEGF, collectively promoting fibroblast proliferation and angiogenesis [31,32]. Clinical trials have shown that CA-based formulations significantly accelerate healing of chronic diabetic ulcers and reduce scar formation compared with conventional treatments. Topical Centiderm^®^ (derived from CA) produced faster burn healing than 1% silver sulfadiazine due to its high antioxidant and antibacterial activity [33]. Polymeric hydrogels incorporating CA extracts demonstrated strong in vitro anti-inflammatory and regenerative performance, achieving complete scratch closure with high fibroblast compatibility. Further in vivo validation remains essential to confirm their full therapeutic efficacy [34,35].

*Calendula officinalis* (Marigold) extracts contain terpenoids, flavonoids, phenolic acids, carotenoids, and coumarins, which contribute to its anti-inflammatory, antioxidant, and immunostimulant activities. Their anti-inflammatory effects are mediated by downregulation of IL-6 and TNF-α, while triterpenoids play a key role in enhancing fibroblast migration during the early stages of tissue repair [36]. When incorporated into poly(vinyl alcohol)-based hydrogels as hydroglycolic extracts, *calendula* promoted early re-epithelialization and increased collagen content after 21 days in rat models compared with unloaded hydrogels. Gamma-irradiated crosslinked formulations retained both biocompatibility and enhanced healing effects [37].

Several kinds of active compounds, such as chamazulene, alpha bisabolol, bisabolol oxides, spiroethers, and flavonoids, are responsible for the therapeutic benefits of *Matricaria chamomilla* L. (chamomile). In comparison to corticosteroids, aqueous and topical extracts of chamomile accelerate wound healing in rats, exhibiting an enhanced rate of wound contraction, increasing tissue strength, and a quicker recovery. Furthermore, chamomile extracts exhibit anti-inflammatory, immunosuppressive, and immunomodulatory properties. The anti-inflammatory effects are mediated through COX-2 inhibition and reduction of nitric oxide production via downregulation of iNOS [38]. Starch/zeolite/chamomile hydrogels demonstrated complete wound closure in 21 days in animal studies, characterized by enhanced epithelialization, collagen formation, and reduced inflammation relative to controls. In clinical applications involving patients with traumatic wounds treated with 2 wt% chamomile hydrogel, the average healing time was approximately 31 days with no reported infections or adverse reactions, underscoring the safety and efficacy of the formulation [39].

The bioactive components of *Hypericum perforatum* L. (HP) extract such as naphthodianthrones, phloroglucinols, flavonoids, bioflavonoids, and phenylpropanoids, exhibit antifungal, anti-inflammatory, antimycobacterial, and antiviral properties, and stimulate collagen synthesis, fibroblast proliferation, and revascularization, which facilitate wound healing. These effects are partly mediated through modulation of inflammatory cytokines and the partial inhibition of protein kinase C (PKC) signaling pathways [40]. When incorporated into Ultrez-based hydrogels (Ultrez 21/30), nanoemulsions containing HP significantly enhanced wound closure in rabbits compared with both macerated extracts and commercial control gels. Complete healing occurred within 12 days, supported by dense collagen formation and re-epithelialization, confirming its strong regenerative potential [41].

### 2.2. Bee-Derived Products

Honey and propolis are highly effective bioactive ingredients for hydrogel dressings due to their multifactorial mechanisms of action. Honey exhibits strong antioxidant activity mediated by enzymes such as catalase, superoxide dismutase, and glutathione peroxidase [42], antimicrobial effects driven by flavonoids and phenolic acids [43], and pronounced anti-inflammatory and moisturizing properties that enhance granulation tissue formation and angiogenesis [44]. Incorporation of honey into polymeric hydrogels has yielded accelerated healing in in vivo models: sodium-alginate (SA)/honey hydrogels containing 4% Dabur honey achieved complete closure of 1 cm wounds within 12 days [45], while chitosan-based hydrogels with 75% Manuka honey healed 10 mm wounds in 9 days [46].

A comprehensive overview of the main natural and bee-derived extracts incorporated into hydrogel-based wound dressings, along with their bioactive compounds and corresponding therapeutic effects, is provided in Table 1, which consolidates the experimental evidence presented above.

**Table 1:** Natural extracts and bee-derived products used in hydrogel-based wound dressings.

Natural Extracts	Plant Source	Bioactive Compounds	Wound–Healing Properties	Wound-Healing Applications
Aloe vera [47]	*Aloe vera*	Mannose-rich polysaccharide (glucomannan), anthraquinones, vitamins B1/B6/ B12, gibberellin, growth hormones	Anti-inflammatory, antimicrobial, antioxidant, stimulates fibroblast proliferation, collagen synthesis, and angiogenesis	Enhances wound closure, hydration, and collagen deposition when incorporated into hydrogel matrices
Curcumin [48]	*Curcuma longa* (Turmeric)	Curcumin (polyphenolic compound)	Anti-inflammatory, antioxidant; promotes collagen deposition, wound contraction, and angiogenesis	Controlled release from hydrogels enhances healing efficacy
Neem [49]	*Azadirachta indica*	Nimbidin, terpenoids, flavonoids, azadirachtin	Antibacterial, antifungal, antiviral, anti-inflammatory; supports tissue regeneration	Promotes fibrocollagenic tissue formation and accelerates wound repair
Green tea [50]	*Camellia* *sinensis*	Epigallocatechin gallate (EGCG), catechins, polyphenols	Antimicrobial, antioxidant, anti-inflammatory; stimulates keratinocyte proliferation and skin regeneration	Provides antimicrobial and pro-regenerative effects in hydrogel formulations
Centella [51]	*Centella* *Asiatica*	Asiatic acid, madecassoside, triterpenoids	Anti-inflammatory, antimicrobial; promotes fibroblast proliferation, ECM and collagen synthesis, tissue regeneration	Facilitates dermal repair and skin regeneration in hydrogel systems
Calendula [52]	*Calendula* *officinalis* (marigold)	Flavonoids, saponins, triterpenes (faradiol monoesters), carotenoids, tannins	Anti-inflammatory, antioxidant, antimicrobial; enhances fibroblast proliferation and re-epithelialization	Exhibits anti-inflammatory and pro-healing effects in wound dressings
Chamomile [53]	*Matricaria chamomilla*	Bisabolol, chamazulene, apigenin, luteolin	Anti-inflammatory, antioxidant, antispasmodic; accelerates burn and wound healing	Provides anti-inflammatory and healing-enhancing activity in hydrogels
St. John’s Wort [50]	*Hypericum perforatum*	Hypericin, hyperforin, flavonoids	Antimicrobial, anti-inflammatory, antioxidant; supports tissue repair	Enhances antimicrobial protection and promotes tissue regeneration
Honey/ Propolis [54]	Honey, bee-derived products	Flavonoids, phenolic acids, organic acids, enzymes (glucose oxidase), vitamins	Broad-spectrum antimicrobial (bacteriostatic/bactericidal), anti-inflammatory, antioxidant; stimulates VEGF expression and fibroblast proliferation	Promotes wound closure and tissue repair through occlusive and bioactive effects

Comparatively, curcumin, green tea, and *Centella asiatica* exhibit the most potent pro-angiogenic effects through upregulation of VEGF, whereas *Aloe vera*, calendula, and chamomile provide broader anti-inflammatory coverage. *Azadirachta indica* (Neem) and *Hypericum perforatum* demonstrate the most extensive antimicrobial spectra, particularly against Gram-positive bacteria. Meanwhile, bee-derived products such as honey and propolis offer a well-balanced combination of antimicrobial, antioxidant, and regenerative properties. These distinctions underscore the unique contributions of each extract to wound healing and highlight the importance of selecting specific agents based on the desired therapeutic mechanism.

## 3. Formulation and Synthesis Strategies of Hydrogels Incorporating Natural Therapeutic Agents

### 3.1. Extraction and Purification of Natural Therapeutic Agents

**Figure 2:**
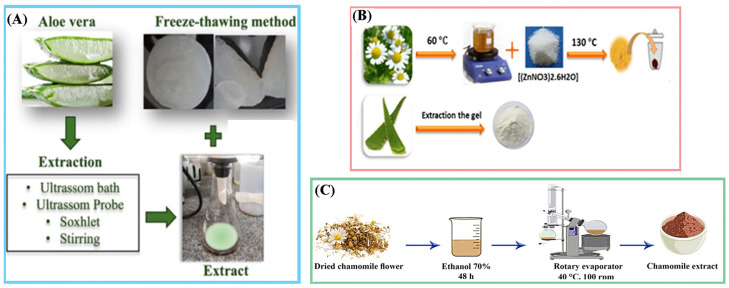
(**A**) Extraction processes of Aloe vera leaves; (**B**) Preparation of extracts of *Matricaria chamomilla* and *Aloe vera* powder; (**C**) Extraction of Chamomile. Adapted with permission from refs. [61,75,96].

The preliminary stage of *Aloe vera* extraction involves washing, drying, peeling, and grinding the leaves, sometimes followed by disinfection with 1–2% sodium hypochlorite for 30 min and thorough rinsing to remove residual disinfectant [55]. The basal portions of *Aloe vera* leaf should be avoided, as they contain anthraquinones and glycosides, bioactive compounds that may cause allergic reactions when applied directly to the skin [56]. Leaves are typically cut to 0.5 mm, frozen at −18 °C for 24 h, and freeze-dried at −55 °C for 72 h, after which the product is ground and stored in amber vials under refrigeration. Various extraction methods are then applied:

**(1)** Stirring and maceration uses 1 g of *Aloe vera* with 25% ethanol at 60 °C and 180 rpm for 1 h, followed by centrifugation (4 °C, 9000 rpm, 10 min), washing of the residue, rotary evaporation (60 °C, 370 mbar, 50 rpm) to remove ethanol, and freeze-drying for 70 h to obtain the extract [57];

**(2)** Precipitation methods include (NH_4_)_2_SO_4_ addition to crude gel to remove proteins, followed by dialysis and lyophilization [58], or mixing *Aloe vera* aqueous extract with ethanol (5:1, *v*/*v*) to flocculate high-molecular-weight, alcohol-insoluble polysaccharides, which are then separated, washed, resuspended, frozen, and lyophilized [59];

**(3)** Soxhlet extraction employs 5–10 g of lyophilized *Aloe vera* with solvents such as ethanol, petroleum ether, or 98% n-hexane/2% ethanol [60] at reflux for 6–8 h, with no yield improvement beyond 8 h, and the extract is concentrated at 60–80 °C under reduced pressure [61,62];

**(4)** Ultrasound-assisted extraction can use either a bath or probe system; the bath extraction is faster, requires <5 g of powder, and less solvent, while the probe extraction requires more material, operates at 500 W and 20 kHz with 30% amplitude (Figure 2A) [61];

**(5)** Supercritical CO_2_ extraction is a clean, safe, and non-toxic method requiring >45–50 g of lyophilized powder, achieving higher yields than ultrasound-assisted extraction [63]. It can be performed under mild conditions (10 MPa, 313 K, CO_2_ flow 0.15 kg/h) or under higher pressures (30 MPa, 373 K) to obtain antioxidant-rich extracts [64]. Its non-toxicity and non-mutagenic effect on the *Aloe vera* gel have been demonstrated by Tanaka et al. [65];

**(6)** Microwave-assisted using very small amounts of *Aloe vera* (~50 mg), usually from fresh smashed leaves mixed with double-distilled water. The mixture is heated in a domestic-type microwave oven (2.45 GHz, 80 W) equipped with a water-cooled condenser to prevent solvent loss. Extraction proceeds in cycles of 1 min “on” and 30 s “off” [66].

The *Calendula officinalis* flower (air-dried and ground) was macerated with 50–70% *v*/*v* ethanol for 5–10 days at room temperature [67], and another method using a plant-to-solvent ratio of 1:10 (g/mL) [68]. Using percolation extraction, ~1 kg of plant is macerated in 70% hydroethanolic solution for 72 h, followed by percolation for 5 days [69]. By glycolic extraction, ~10 g of flowers is macerated in polyethylene glycol–water solution (9:1) in an amber glass flask at room temperature for 14 days with intermittent stirring [70]. Soxhlet extraction of *C. officinalis* uses ~500 g of air-dried and powdered flower, temperature of 70–80 °C for 8 h, employing n-hexane and ethanol as solvents [71].

*Matricaria chamomilla*, commonly known as chamomile, is typically incorporated into hydrogels through infusion extracts prepared at 80 °C [72] or at 60 °C, as shown in Figure 2B, hydroalcoholic extracts obtained via sonication at 400 W and 20–24 kHz in on/off cycles [73], or Soxhlet extraction [74]. Additionally, maceration is performed using 70% *v*/*v* ethanol for 48 h at 25 °C (Figure 2C) [75].

*Centella asiatica* can be extracted using several methods. One approach involves maceration with 95% ethanol (1:3 *w*/*v* ratio) for three days [76,77]. Alternatively, fresh leaves and rhizomes may be juiced and centrifuged at 4000 rpm [78]. Another method employs ultrasound-assisted extraction with 70% methanol as the solvent at 70 °C for three cycles of 60 min [35].

Curcumin is extracted from the rhizomes of *Curcuma longa* and is commercially available as a reagent with a purity ranging from 95% to 98%, supplied by various specialized chemical reagent companies. In addition, synthetic curcumin, with the molecular formula C_21_H_20_O_6_ and a purity greater than 97%, is also available on the market.

Grape seeds are extracted from the grape skins by filtering the mixture through a mesh sieve. The process of preparing grape seed powder involves a series of steps: washing, drying, sterilization, freezing, and superfine grinding [79]. An alternative of grape seeds extraction procedure includes using 33% (*v*/*v*) acetone in water for 15 h at room temperature and in darkness to prevent oxidation [80].

The extraction of green tea is performed using dried leaves in water or boiling water (80–95 °C, 10–60 min), followed by filtration and centrifugation (5000–8000 rpm, 10 min), and subsequently drying in a hot air oven at 60 °C or through lyophilization at −60 °C, resulting in a dark brown aqueous extract of green tea [81,82,83].

Neem leaf extract is obtained from the leaves of *Azadirachta indica*. Approximately 20 g of chopped leaves, finely powdered, are suspended in 100 mL of double-distilled water (DDW). The mixture is subjected to heating (70–80 °C, 30 min) or boiling at 100 °C (2 min). The resulting solution is filtered using a 0.45 μm PVDF Millex filter [84,85]. Another method involves using 100 g of finely ground dried leaves in distilled water with continuous shaking for 5–7 h at 120 rpm. The extract is filtered with Whatman No. 1 paper and subsequently lyophilized. The extract is reconstituted in purified water at different concentrations [25,86]. To prepare a macerate of neem leaves, a crude powder of mashed leaves is treated with 70% ethanol for 7 days. The filtrate is then concentrated using a rotary evaporator at 50 °C to yield a viscous extract [87].

The formulation of the hydrogels incorporated various types of honey, including manuka, monofloral honey, clove, cotton, fennel flower, lemon, orange, rapeseed, buckwheat, linden and vegetable honeys [44,88]. The concentrations of honey used were 0%, 5%, 10%, 15%, and 20%, or even 400% (*w*/*w*) [89]. The raw propolis material is obtained from local beekeepers, with concentrations varying from 1% to 5% (*w*/*v*). Alcoholic propolis is obtained by extracting with 50–80% ethanol at room temperature for 24 h, followed by centrifugation to eliminate the wax present in the propolis [90]. Other studies used a 30% (*w*/*v*) propolis extract in water, diluted to a concentration of 2% (*w*/*v*), which is considered optimal for cosmetic applications aimed at anti-inflammatory effects [91]. Additionally, the propolis tincture can be obtained through maceration, using 200 g of raw propolis and 2 L of 70% ethanol, stored in amber bottles at room temperature for 7 days [92].

Bioactive extracts of St. John’s wort (*Hypericum perforatum* L.) are obtained through hot water extraction at 85 °C for 5 h with stirring at 800 rpm. The resulting extract is then concentrated using a vacuum rotary evaporator at 60 °C for 1 h, followed by drying in a vacuum concentrator at 45 °C for 24 h [93]. *Hypericum perforatum* L. seeds are sterilized and germinated on a medium containing 2% sucrose and 0.75% agar (pH 5.6–5.8) at 25 ± 2 °C under a 16 h light/8 h dark cycle. Seedlings (3–5 mm) are introduced to callus-inducing medium with 2,4-D, BAP, 2% sucrose, and 0.75% agar. The dried callus is extracted with 100 mL of 80% ethanol for 72 h, centrifuged (14,000 rpm, 4 °C, 15 min), filtered, and concentrated under vacuum, freeze-dried for 48 h, and stored at −20 °C [94,95].

### 3.2. Incorporation Strategies for Natural Therapeutic Agents

#### 3.2.1. Direct Mixing (Before Crosslinking)

Direct incorporation of natural therapeutic agents into polymer precursor solutions prior to the crosslinking stage is one of the most straightforward and efficient strategies for hydrogel formulation [97]. Such loading approaches are widely applied across diverse polymeric and composite hydrogel systems, which possess hydrated networks and tunable crosslinking that enable efficient encapsulation and controlled release of bioactive molecules. In this context, recent literature also highlights hydrogels as a representative class of biomimetic matrices, whose viscoelastic structure and adjustable chemistry support cell adhesion, modulate inflammation, and promote wound healing [98,99]. This approach allows a homogeneous dispersion of bioactive compounds throughout the polymeric matrix, ensuring controlled distribution and enhanced therapeutic performance after gel formation.

Hanif et al. prepared composite hydrogels by dissolving polyvinyl alcohol (PVA), graphene oxide (GO), and *Aloe vera* extract in demineralized water, followed by ultrasonic homogenization and repeated freeze–thaw cycles to achieve uniform distribution of the extract throughout the hydrogel network [100]. Another study mixed *Aloe vera* extract with a 10% (*w*/*v*) PVA solution maintained at 50 °C, together with caffeine, vitamin C, and gentamicin prior to crosslinking; insolubility was induced by soaking the membranes in propyl alcohol, allowing simultaneous incorporation of multiple bioactives at the precursor stage [101]. In another study, silk fibroin (SF) hydrogels were produced by combining dialyzed fibroin solution with *Aloe vera* extract (3:1 *v*/*v*), followed by freezing and thawing cycles to induce gelation, resulting in hydrogels with a uniform microstructure [61]. Similarly, CS/PVA hydrogels were fabricated by blending polymer solutions and adding *Aloe vera* extract (5–15 wt%) and ZnO nanoparticles under continuous stirring before alkaline crosslinking [96]. Further, porous CS/SF/AV scaffolds were obtained by combining *Aloe vera* powder with chitosan and silk fibroin in specific ratios, followed by freezing and lyophilization [58].

*Calendula officinalis* extract, stabilized with glycerol and TWEEN-80, has been incorporated into polymeric matrices by direct blending at concentrations up to 5% relative to a carboxymethylcellulose (CMC)-PVA mixture, with one formulation containing the extract/glycerol (Gly)/TWEEN-80 in a 95:1:4 (*v*/*v*) ratio [102]. Other approaches include mixing of glycolic *Calendula* extract (10% *v*/*v*) with a 1.5% alginate–1% CMC precursor solution [70] and preparing PVA/carrageenan (κ-CAR) hydrogels with hydro-glycolic *Calendula* extract at moderate temperatures prior to gamma irradiation [37].

**Figure 3:**
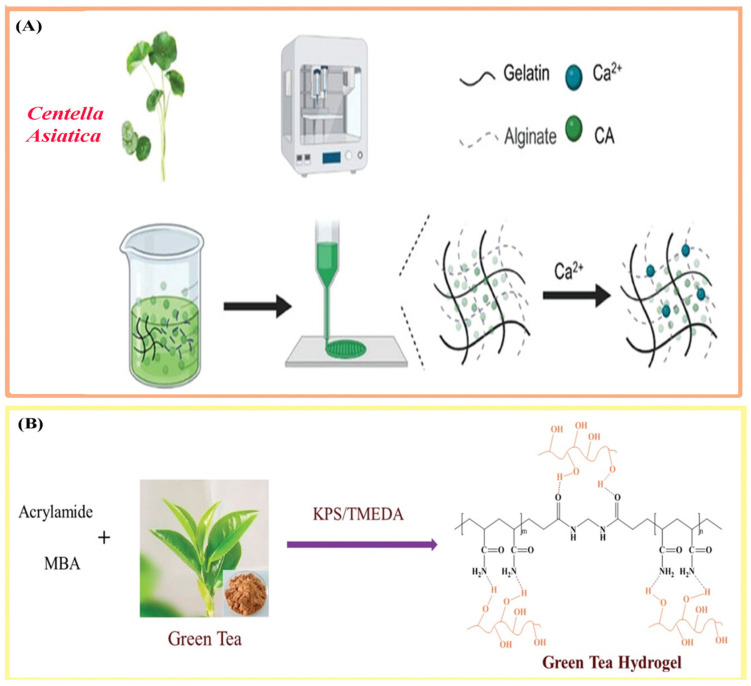
(**A**) Preparation of *Centella asiatica*@3D scaffold in wound healing; (**B**) Preparation of Green tea hydrogels. Adapted with permission from refs. [78,116].

*Centella asiatica* has been directly incorporated into various polymeric matrices using several formulation strategies. For instance, the asiaticoside-rich fraction (24 mg) was dissolved into autoclaved PVA/PEG precursor solutions prior to the freeze–thaw processing step [103]. In another study, CA extract was uniformly mixed into carbomer-based hydrogels below 40 °C under moderate stirring to preserve the bioactive components [77]. Furthermore, gelatin-based systems were formulated by adding CA extract to aqueous gelatin, followed by the addition of CS and pH adjustment to ensure a homogeneous precursor solution [35]. Similarly, gelatin/SA compositions were prepared using gelatin and SA combined with CA extract, resulting in a uniform biopolymer network (Figure 3A) [78].

In addition, chamomile extract (10% *w*/*w*) was dispersed directly uniformly into sodium alginate/gelatin mixtures prior to genipin and CaCl_2_ crosslinking [75]. Likewise, neem-mediated ZnO nanoparticles were added under stirring to a CS solution crosslinked with triethyl orthoformate, allowing 16 h dispersion at room temperature [104].

Moreover, honey-based hydrogels have been developed using different combinations of natural polymers and additives. Manuka honey was blended with CS and gelatin in equal proportions to obtain bioactive wound dressings [44], whereas in another formulation, honey (20% *v*/*v*) and *Aloe vera* (2.5% *v*/*v*) into alginate–CS solutions before ionic crosslinking with CaCl_2_ [105]. Additionally, formulations combining *Aloe vera* (1%) and honey (0–20%) with PVA were crosslinked with borax, leading to flexible and transparent hydrogel films [89]. Propolis has also been introduced into mung bean protein/κ-CAR matrices by dissolution in ethanol, followed by mild heating to gelation [106]. Similarly, honey-enriched hydroxyethyl cellulose hydrogels were obtained through sequential mixing of honey, glycerine, and polymer under gentle heating conditions [88]. Further, natural polysaccharides such as agar, SA, and apple pectin were directly dissolved in plant extracts under heating or autoclaving conditions, followed by ionic crosslinking with CaCl_2_ to produce biopolymeric networks [93]. Finally, *Hypericum perforatum* callus extract was incorporated into CS–alginate mixtures at varying concentrations (0–2000 μg/mL) prior to CaCl_2_ crosslinking and lyophilization, yielding porous and biocompatible hydrogels with tunable phytochemical loading [95].

#### 3.2.2. In Situ Incorporation (During Gelation)

In situ incorporation of natural therapeutic agents involves the introduction of bioactive compounds directly into the polymerization or gelation stage, enabling their entrapment or chemical integration within the developing hydrogel network. This approach ensures a uniform distribution of the natural extract during network formation and reduces phase separation or losses during post-processing [107].

Shanmugha Mary et al. synthesized a series of hybrid hydrogels based on gelatin (1–5% *w*/*v*, crosslinked with 25% glutaraldehyde), alginate (2% *w*/*v*, crosslinked with 1 M CaCl_2_), starch (10:1 ratio with urea), and PVA (12% *w*/*v*), in which *Aloe vera* extract (5 g dried powder in 100 mL water) replaced conventional Milli-Q water during polymer dissolution [108]. In another study, composite gelatin methacryloyl (GelMA)-based hydrogels were obtained by mixing GelMA (40% *w*/*v*), DMA (5%), Irgacure 2959 (5%), NaOH (0.5% *w*/*v*), and 100% *Aloe vera* extract in equal proportions, followed by UV photocuring (100 mW/cm^2^, 5 min), ensuring uniform incorporation during photopolymerization [109]. Moreover, concentrated *Aloe vera* extract, pasteurized and filtered, was incorporated into poly(NVP-co-acrylamide) matrices (molar ratio 2:3). After partial copolymerization via Fenton’s reagent, *Aloe vera* (0–12% relative to polymer weight) was added prior to MBA crosslinking, leading to homogeneous hybrid networks [110].

Similarly, CA extract was introduced into precursor solutions containing CS, poly(β-amino ester) (PβAE), and the photoinitiator 2,2-dimethoxy-2-phenyl-acetophenone (DMPA) prior to UV exposure, ensuring even dispersion within the polymer matrix [111]. In a comparable approach, chamomile extract was integrated into polymerization mixtures containing neutralized acrylic acid (40% KOH), starch (cold or hot), PEG diacrylate, and a photoinitiator system, facilitating uniform network formation after photo-crosslinking [72].

Curcumin has also been extensively employed through in situ methods across multiple hydrogel systems. For example, curcumin, nanocellulose, and Tween 20 were dispersed directly into a CS solution (5% acetic acid) under stirring, followed by glutaraldehyde crosslinking to yield uniform CS/nanocellulose composites [112]. In another case, curcumin (1.2–7.2 mg/mL in DMF) was introduced into a Z-Tyr-Phe-OH dipeptide precursor solution before gelation, producing stable hybrid gels with evenly distributed bioactive molecules [113]. Furthermore, modified quaternized chitosan hydrogels were prepared by adding curcumin (2 wt%) to the pre-gel solution prior to pH-induced self-gelation (pH 7.5), resulting in stable hydrogels with encapsulated antioxidant activity [114]. In protein-based systems, curcumin was blended with protein–sorbitol solutions during the heating process, followed by ultrasound treatment to enhance molecular dispersion before gel formation [115].

Plant extracts have also been successfully incorporated during polymerization in polyacrylamide (PAAM)-based networks. For instance, hydrogels were synthesized by dissolving acrylamide and green tea extract (0.0025–0.01 g) in water, followed by the addition of MBA crosslinker and a KPS/TMEDA initiator system (Figure 3B) [116]. Similarly, *N*-isopropylacrylamide (NIPAM) was copolymerized in green tea aqueous solution (0.0054 mM) using the same initiator system, forming P(NIPAM–GT) hydrogels enriched with catechin components [117]. Another formulation combined green tea extract and gelatin in alkaline medium (pH 11), followed by crosslinking with sodium periodate, triggering sol–gel transition and generating antioxidant hybrid networks [81].

In situ strategies were further extended to the preparation of neem- and propolis-based hydrogels. Neem gum was dissolved in water under stirring, followed by the sequential addition of ammonium persulfate initiator, acrylamide monomer (AAm), and MBA crosslinker [118]. Similarly, propolis extract (1.0–2.5%) was incorporated into a poly(acrylamide)–methylcellulose precursor solution prior to the addition of sodium persulfate and TEMED, resulting in transparent hydrogels containing the natural antimicrobial agent [92].

#### 3.2.3. Post-Loading (After Gel Formation)

Post-loading involves the incorporation of natural therapeutic agents into preformed hydrogel matrices after network formation. In this approach, bioactive compounds diffuse into the dried or swollen hydrogel structure via immersion or soaking, allowing adsorption, physical entrapment, or ionic interaction without disrupting the crosslinked polymer framework. This approach is especially suitable for thermosensitive or photosensitive compounds prone to degradation during synthesis [119].

Ghasemi et al. reported the loading of *Calendula officinalis* flower extract into preformed CS/sodium alginate/PVA hydrogels by immersion for 2 h, enabling diffusion of the active components into the crosslinked network [120]. Similarly, *Calendula officinalis* hydroalcoholic extracts (5–20% *w*/*v*, ethanol/water 50:50 *v*/*v*) were introduced into polypropylene/PVA hydrogels through soaking, followed by air-drying and lyophilization to ensure stable entrapment of the extract and preservation of antioxidant capacity [121]. In another study, *Calendula* extract (1 mg/mL) was diffused into okra mucilage/2-acrylamido-2-methylpropane sulfonic acid hydrogels for 24 h, followed by vacuum drying, achieving homogeneous loading within the polymeric matrix [122].

Post-loading has also been applied to the CA extract in various hydrogel systems. Hybrid hyaluronic acid (HA)–dextran patches were impregnated with CA extract at weight ratios ranging from 9.8:0.2 to 9:1, then washed and lyophilized to ensure uniform incorporation [123]. In another formulation, CA extract was loaded into gelatin/Fe-MOF and methacrylated gelatin/Fe-MOF hydrogels by immersing dried samples (0.15 g) in 50 mL of extract solution (250 ppm) for 24 h [124]. Likewise, dried hydrogels were soaked in CA extract solutions for 24 h and dried at 40 °C, facilitating efficient phytoconstituent diffusion [125].

Furthermore, curcumin has been post-loaded into gelatin-based hydrogel films via immersion in a 5 mg/mL DMSO curcumin solution at room temperature, enabling effective drug diffusion and entrapment within the gelatin network [126]. A similar strategy was adopted for PVA–grape seed extract–ammonium sulfate hydrogels, in which the crosslinked network was first formed via freeze–thaw cycles, followed by immersion in 40 wt% ammonium sulfate solution to post-load the salt [127]. Moreover, green-tea-enriched hydrogels were obtained by loading a green tea extract/β-cyclodextrin inclusion complex (10–50 wt%) into CS/PVA hydrogels after gelation, achieving uniform antioxidant dispersion within the hybrid matrix [128]. In another study, *Azadirachta indica* (neem) extract (2 g) was dissolved in 0.1 M HCl and combined with preformed CS hydrogel, followed by nanoparticle addition and 24 h stirring, resulting in efficient post-loading and crosslinking within the structure [129].

Sharaf et al. introduced honey bee propolis extract (4%) into κ-CAR/β-cyclodextrin hydrogels applied onto textiles, ensuring homogeneous distribution by mild heating at 60 °C [130]. Finally, gelatin/xanthan gum (XG) hydrogels, thermally crosslinked at 130 °C for 30 min, were soaked in 50% *w*/*w* honey solution for 1 h, yielding honey-loaded patches with uniform absorption throughout the matrix [131].

#### 3.2.4. Nano/Microencapsulation

Nano- and microencapsulation represent advanced strategies for incorporating natural therapeutic agents into polymeric hydrogels, providing enhanced stability and protection against degradation of bioactive compounds. In these approaches, plant extracts or active phytoconstituents are first encapsulated within nano- or microcarriers, such as liposomes, polymeric nanoparticles, or ionically crosslinked beads, before being integrated into the hydrogel matrix [132].

Julie et al. developed a composite hydrogel system by encapsulating *Aloe vera* and *Curcuma longa* extracts via ionic gelation using 0.6% SA and 1% CaCl_2_, followed by freeze-drying at −40 °C and 0.5 bar. The resulting microcapsules were subsequently embedded within gelatin (1%), polyethylene glycol (PEG) (3%), and CS (1%) hydrogels crosslinked with 0.5 M NaOH, yielding a system capable of enhanced stabilization of the encapsulated bioactives [133]. Likewise, *Aloe vera* extract was encapsulated within calcium alginate or alginate–gelatin hydrogel beads by mixing the extract with alginate solutions and crosslinking with CaCl_2_ or glutaraldehyde. This process produced stable microbeads with well-dispersed bioactive content suitable for hydrogel incorporation [134]. In a related contribution, chamomile extract was nanoencapsulated via the preparation of a microemulsion composed of chamomile extract and almond oil (1:3 *v*/*v*) stabilized with Triton X-100, which was then dispersion in a 1% tragacanth gum (TG) solution and sonication [73].

Numerous studies have focused on curcumin encapsulation to overcome its hydrophobicity and poor solubility. One formulation strategy involved the creation of a Curcumin/Pluronic^®^ complex by dissolving curcumin in ethanol and blending it with an aqueous Pluronic^®^ solution, followed by the addition of PVA and mild heating at 50–70 °C. The curcumin/Plu-loaded PVA mixture was subsequently combined with TEMPO-oxidized cellulose nanofibers (TOCN), resulting in nanostructured hybrid hydrogels with improved molecular dispersion and stability of curcumin [135]. Another investigation incorporated liposomal curcumin dispersion into preformed Carbopol hydrogels containing lysine and collagen, achieving nanoscale homogeneity and long-term stabilization of the active compound within the crosslinked network [136]. Additionally, curcumin-loaded nanoemulsions were combined with alginate-based polymer solutions (CS, gelatin, or polyethylene oxide (PEO)) at a 1:3 ratio and subsequently dripped into 2% CaCl_2_ solution to generate ionically crosslinked microbeads. These spherical beads exhibited highly uniform curcumin loading [137]. Furthermore, nanoparticles were obtained by dissolving curcumin in aqueous 1% sodium dodecyl sulfate and introducing this solution dropwise into CS solution (0.12% *w*/*v* in 4% acetic acid). The subsequent pH adjustment to 4.76 using trisodium citrate induced ionic complexation between curcumin and chitosan, leading to spontaneous nanoparticle self-assembly with high encapsulation efficiency [138].

Collectively, the studies summarized in this section illustrate that the choice between direct mixing, in situ incorporation, post-loading, and nano/microencapsulation is primarily dictated by extract stability, processing temperature, and polymer crosslinking requirements, rather than by differences in healing efficacy.

## 4. Biological Evaluation of Hydrogels Containing Natural Therapeutic Agents

### 4.1. Natural Therapeutic Extracts in Wound Repair: Experimental Insights from In Vitro and In Vivo Models

This section summarizes the experimental findings compiled in Table 2 and Table 3, which detail hydrogel formulations incorporating natural extracts evaluated for wound-healing applications. Both tables provide comprehensive data on the polymeric composition of the hydrogels along their therapeutic or biological effects outcomes reported in the literature. Table 2 contains standardized natural extracts with defined compositions, whereas Table 3 presents non-standardized or crude extracts obtained through diverse extraction methods. The biocompatibility and wound-healing efficacy reported in these studies were evaluated using well-established in vitro and in vivo methodologies, including assays for fibroblast viability and migration, hemocompatibility analyses, wound closure measurements, histological examinations, as well as assessments of collagen deposition and neovascularization.

**Table 2:** Standardized natural and bee-derived extracts in hydrogel-based wound dressings.

Natural Agents	Type of Standardization	Polymeric Composition	Therapeutic & Biological Effects
*Aloe vera*	Powder extract (200× concentrate) [100]	10% PVA–0.05% GO	Exhibits antibacterial activity (99.94% inhibition *S. aureus*); Enhances fibroblast viability (~295%), and demonstrates excellent cytocompatibility.
	Powder extract [139]	2% Alginate–0.01% COL–0.1% Gly	Promotes Hs27 fibroblast proliferation (up to 165%); Antibacterial activity (75–90% reduction) against *S. aureus*, *E. coli*, and *P. aeruginosa*; Enhanced wound healing potential.
	Powder extract [59]	1% Carbopol 940–2% HEC	Demonstrates effective anti-inflammatory and skin regenerative properties.
	Powder extract (purity of 99.01%) [140]	50% Gelatin–20% Agar–10% Gly	Non-cytotoxic to HDF and HaCaT cells; Promoted cell adhesion, migration, and wound closure; Exhibits anti-inflammatory activity.
	Powder extract [141]	0.1% CS–0.1% dextran sulfate (DXS)	Exhibits antibacterial activity (≈50% inhibition of *S. aureus* and *S. mutans*); Shows antioxidant potential and improved wound-healing efficacy.
	Powder extract [108]	2% Alginate–5% Gelatin	Demonstrates antibacterial activity against *P. aeruginosa* (inhibition zones ≈ 15.4 ± 3 mm for gelatin and 13.7 ± 2 mm for alginate hydrogels).
	Powder extract [109]	5% PDMA–8% GelMA	Provides superior wound-healing and anti-inflammatory effects; Promotes tissue regeneration and exhibits excellent mechanical and adhesive properties.
*Calendula officinalis*	Hydroalcoholic extract [142]	Carbomer 940–PG–TEA	Effectively manages 5-fluorouracil (FU)-associated hand–foot syndrome.
	70% EtOH macerated extract [68]	Carbopol 980NF–PEG400	Non-biological performance.
	70% EtOH macerated extract [143]	2% CS–25% PEG400	Exhibits antibacterial activity against *P. aeruginosa*; Synergistic effects when combined with other extracts.
	3% Lyophilized hydroalcoholic extract [144]	CS–HPMC (2–3%)	Demonstrated antibacterial activity against *S. aureus*, *P. acnes*, *E. coli*.
	10% Hydroglycolic macerated extract [70]	1.5% SA–1% CMC	Non-cytotoxic effects to 3T3 cells, Promote cell proliferation; Supports cellular processes essential for skin repair.
	50% EtOH macerated extract [145]	XG–PEG 8000	Increases fibroblast migration; Promotes extensive re-epithelialization within 9 d without scarring or hair loss.
	(2–4)% Hydro-glycolic Extract [37]	(8–10)% PVA–(0.75–1.25% κ-CAR	No in vitro hemolysis; Swiss 3T3 albino fibroblasts cells viability above 95%; Increased wound closure after 48 h; Improvement in wound re-epithelialization, neovascularization and wound retraction.
	EtOH extract [122]	Okra mucilage–AMPS-EGDMA	Exhibits antibacterial activity against *S. aureus* and *E. coli*; Increased blood clotting index; Improves wound closure and overall healing.
	Aqueous extract [146]	(0.5–2.5)% CS–1%PVA	Strong antimicrobial activity against *E. coli*, *S. aureus*, and *C. albicans*; >80% cell viability in L929 fibroblasts; Potential for biomedical and wound healing applications.
*Centella* *asiatica*	EtOH extract [123]	1% HA–9% Dextran	Enhances fibroblast viability and migration at optimal 0.4 wt% CA concentration; Promoted wound closure without cytotoxic effects.
	90% MeOH extract [147]	0.5% CS–1.25% Gelatin	Stimulates fibroblast proliferation (up to 142%); Antibacterial activity against *P. acnes* (26 mm inhibition zone, MIC = 150 µg/mL) and *S. aureus*; Non-cytotoxic and non-irritant, suitable for anti-acne and wound healing applications.
	Aqueous extract [78]	2.5% SA–8% Gelatin	Accelerates wound healing and enhances neovascularization at the wound site; Increases epithelial thickness and hair follicle density; Effective in diabetic chronic-wound repair.
	70% MeOH extract [34]	3% CS	Antibacterial activity against *S. aureus*; Fibroblast migration and wound closure (73.4% at 24 h; 99.0% at 48 h); Synergistic wound-healing efficacy with good biocompatibility.
	70% EtOH extract [124]	3.6% Gelatin–3.6% MAAn	Antibacterial activity (inhibition zones 19–28 mm; MIC = 3.125–6.25 mg/mL) against *Bacillus*, *E. coli*, *S. aureus*, *P. aeruginosa*, *Klebsiella*, *Streptococcus*, and *C. albicans*; Non-toxic, biocompatible and promoted cell growth.
	96% EtOH extract [111]	30% CS–poly (β amino ester)	Achieves 99.5% wound closure within 24 h; Antibacterial activity against *E. coli* and *S. aureus*; Positive effect on L929 cell proliferation.
	Ultrasound-assisted extract (70% MeOH) [35]	3% CS–2% Gelatin	Promotes fibroblast migration and wound closure (up to 99.9% after 36 h); Biocompatible, stimulates collagen synthesis and angiogenesis.
	70% EtOH extract [125]	Gelatin–AA–AAm	HFF-2 cell viability; Significant antibacterial efficacy against multiple bacterial strains.
*Chamomile*	Aqueous extract [148]	0.5% TG–0.5% SA–0.5% BC	Antibacterial activity against Gram-positive and Gram-negative bacteria; Enhanced fibroblast cell viability and proliferation; Non-cytotoxicity and excellent biocompatibility.
*Curcumin* (*Curcuma longa*)	Powder extract [126]	5% Gelatin– (0.5-1%) BC	Antibacterial activity (*E. coli*: 15 ± 0.5 mm; *S. aureus*: 19 ± 1 mm inhibition zones); Induced bacterial cell wall damage and death; Enhanced fibroblast proliferation and migration, leading to complete wound closure with 18 h; Antioxidant and anti-inflammatory effects.
	100% EtOH extract [135]	1% TOCN–(5–10)% PVA	Promoted fibroblast proliferation and collagen deposition; Enhances wound contraction and tissue regeneration; Accelerates re-epithelization (≈81% closure after 2 weeks); Cell viability (~85%) and strong biocompatibility.
	Powder extract [149]	4% SA–20.4% NIPAM	Accelerated wound contraction (≈96.5% closure at day 14) with complete re-epithelialization; Enhanced fibroblast proliferation, collagen deposition, and angiogenesis; Significantly reduces inflammation via NF-κB/TNF-α/IL-1 inhibition; Biocompatibility and regenerative effect.
	Powder extract [150]	0.5% GG	Fibroblast proliferation (+45%) and collagen synthesis (+50% at 14 days); Enhanced cell migration (up to 100% vs. control) and re-epithelialization (73% closure at day 12); Antibacterial activity and excellent biocompatibility (≥80% cell viability).
	MeOH extract [136]	2% Carbopol–0.25% COL	Wound-healing efficacy with 79.25% wound contraction by day 3 and complete closure by day 7, without scarring; Re-epithelialization (>70%) and collagen deposition; Anti-inflammatory effects via TLR4 and NF-κB inhibition. Excellent cytocompatibility and tissue regeneration.
	Powder extract [151]	0.2% CS–4% PVA	Antibacterial activity against *Streptococcus faecalis* and *E. coli* (MIC = 2.60 ± 1.13 µg/mL and 1.30 ± 0.56 µg/mL, respectively).
	Powder extract [152]	3% SF–3% Pluronic F127	Cytocompatibility with fibroblast cells (no cytotoxicity after 1–7 days); Achieving up to 5-log reduction in bacterial growth; Provides potent bactericidal and bioactive properties.
	Powder extract [153]	2% CS–2% Gelatin	Demonstrated >95% antibacterial efficiency against *E. coli* and *S. aureus*; Strong antioxidant performance.
	Powder extract [154]	3.5% Ammonium alginate–3.5% PVA	Antioxidant activity (DPPH = 72.6%; ABTS = 98.50% after 6 h); Excellent biocompatibility (L0-2 cell viability >120% after 24 h) and hemocompatibility (hemolysis <5%).
	Powder extract [114]	2.5% QCS	Excellent cytocompatibility with L929 fibroblasts (cell viability >100% after 5 days; Antioxidant capacity (DPPH scavenging 76.2%); Accelerated wound closure: 63.4% at day 3; 86.6% at day 10); Reduces inflammation and enhances granulation, collagen deposition, and angiogenesis.
	80% EtOH extract [155]	10% GelMA–5% SF	Provides 90.3% (*S. aureus*) and 87.9% (*E. coli*) reduction in colonies; inhibition zones of 30.9 ± 0.55 mm and 32.9 ± 0.49 mm; Maintains fibroblast viability >95% after 3 days; excellent hemocompatibility (hemolysis 1.18 ± 0.34%).
	75% EtOH extract [156]	5–10% GelMA	Excellent cytocompatibility with L929 fibroblasts; Strong antioxidant protection under oxidative stress, restoring H_2_O_2_-treated cell viability; Pronounced anti-inflammatory activity.
*Grape*	70% EtOH extract of white pomace [157]	0.5% CS– 1% Alginate	Moderate antioxidant activity (1.016 ± 0.288 µmol/L); Antibacterial efficacy against *S. aureus* (~97% growth reduction) and non-toxic.
	Draksha–Beeja powdered extract [158]	5% Starch–5% Gelatin	Excellent cytocompatibility with >99% L929 fibroblast viability and strong cell proliferation; Highly hemocompatibility (hemolysis ≈ 1.5%) and regenerative potential.
*Green Tea*	Powder extract [159]	1% PVA–1% SA	Antibacterial activity, stronger against *S. aureus* than *E. coli*; Inhibition halos and OD_600_ reduction confirm effective suppression of bacterial proliferation; Excessive extract loading reduced antimicrobial efficiency.
*Neem* (*Azadirachta indica*)	Aqueous extract [160]	0.25% N-succinyl chitosan–20% Pluronic F127	Demonstrates dose-dependent antioxidant activity in DPPH assay (39% at 0.05 g/mL to 75% at 0.30 g/mL); Potential to reduce oxidative stress and inflammation in wound environments.
	Powder extract [129]	0.1% PCL–0.2% Kolliphor P188	Pronounced antibacterial activity against *S. typhi*, *E. coli*, and *S. aureus*.
*St. John’s Wort*	*H. perforatum* callus powdered extract [95]	1% CS–1% SA	Demonstrates dose-dependent enhancement of fibroblast proliferation; Excellent cytocompatibility (≈96% viability), cell adhesion, spreading, and proliferation; Antibacterial activity against *E. coli* and *K. pneumoniae*.
	80% EtOH extract of *H. perforatum* callus [94]	2% PV–1% CS– 1% Alginate	Excellent cytocompatibility with fibroblast viability around 99%; Enhances cell proliferation up to 150%; Promotes strong cell adhesion and accelerated wound closure (>99% healing within 14 days).
*Honey*	Organic Manuka honey extract [161]	4% CS–4% Gelatin	Exhibits strong antibacterial activity, inhibiting bacterial growth for up to 12 h; Accelerates wound healing via antimicrobial and exudate absorptive effects.
	EtOH extract of Propolis [162]	2% Carbopol 934–2% PG	Antibacterial activity against *S. aureus* and *S. epidermidis*; Excellent cytocompatibility with NIH 3T3 fibroblasts and anti-inflammatory effects; Promotes rapid wound contraction (>90% by day 14) and near-complete re-epithelialization (≈96% by day 28).
	75% EtOH extract of propolis [130]	4% κ-CAR	Antimicrobial activity against *S. aureus*, *P. aeruginosa*, and *C. albicans*; No inhibitory effect against *A. niger.*
	70% EtOH macerated extract of propolis [163]	10% PVA	Antimicrobial activity against *S. mutans*, *E. coli*, and *C. albicans* (inhibition zones up to 19 mm; MIC = 0.025–0.05 mg/mL); Excellent cytocompatibility (≥90% cell viability up to 125 μg/mL) and enhanced fibroblast adhesion.
	70% EtOH macerated extract of propolis [92]	7.2% AAm–0.5% MC	Strong antibacterial activity against *S. aureus* and *P. aeruginosa*; Moderate antifungal activity against *C. albicans* and *C. tropicalis*; Antioxidant potential.

To gain a deeper insight into these results, representative studies were examined in greater detail, emphasizing in vitro and in vivo investigations that elucidate the wound-healing efficacy of hydrogels loaded with natural therapeutic agents. Among the most extensively studied systems, DMA–GelMA hydrogels incorporating *Aloe vera* extract exhibited remarkable regenerative performance. In vitro assays revealed strong stimulation of fibroblast proliferation and migration, with 22.6-fold and 49.5-fold up-regulation of collagen I and III expression, respectively. In vivo, PDMA–GelMA dressings accelerated wound closure, enhanced granulation tissue formation, and increased collagen deposition compared with both the neat hydrogel and a commercial CS/HA biogel [109]. Consistently, multilayer nanofibrous membranes composed of PVA, CS, κ-Car, and *Aloe vera* demonstrated superior healing in burn-injured mice compared with the untreated control. The composition achieved complete wound closure within 28 days, promoting rapid contraction and tissue regeneration without scar formation. Histological analysis revealed reduced inflammation, enhanced fibroblast proliferation, and uniform collagen deposition, accompanied by increased angiogenesis and re-epithelialization produced complete wound closure within 28 days in burn-injured mice. The treated tissue exhibited rapid contraction and regeneration without scarring, accompanied by reduced inflammation, abundant fibroblast infiltration, and uniform collagen deposition, together with intensified angiogenesis and re-epithelialization [164].

The beneficial role of *Calendula officinalis* (marigold) has been confirmed in several independent studies. An alginate-based hydrogel containing 10% (*v*/*v*) glycolic extract of *C. officinalis* promoted 3T3 cell proliferation without cytotoxic effects and significantly accelerated wound contraction in rats by day 14. Histological evaluation revealed diminished inflammatory cell infiltration and enhanced collagen organization, consistent with the anti-inflammatory activity of the extract [70]. Similarly, compositions containing *Calendula officinalis* demonstrated accelerated in vivo wound healing, achieving 96.1 ± 0.9% wound retraction by day 16 for the encapsulated hydrogel, surpassing both the unencapsulated and control groups. The elevated hydroxyproline content (255.7 ± 0.8 µg/mg) and reduced wound index confirmed enhanced collagen formation and rapid tissue regeneration. Histological analysis revealed well-organized collagen fibers and minimal inflammation, confirming the efficacy of the encapsulated Calendula-based hydrogel in promoting wound repair and dermal remodeling [165].

**Figure 4:**
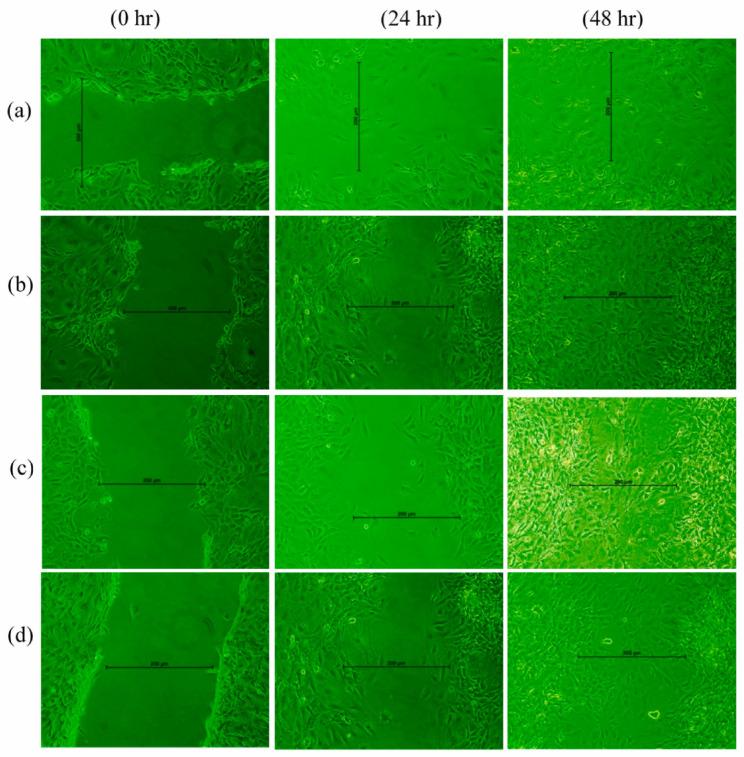
Cell wall after wounding (**a**)—negative control, (**b**)—*C. officinalis* flower extract; (**c**)—Alginate hydrogel; (**d**)—C. officinalis flower extract loaded in hydrogel. Adapted with permission from ref. [37].

A comprehensive investigation on the wound healing properties of *calendula* flower extracts was conducted by applying them to artificially wounded cells (scratch test). Wells treated with the formulation containing *calendula* flower extracts exhibited a greater quantity of fibroblast cells in the scratched region compared to wells treated solely with medium and the placebo hydrogel sheet (Figure 4). Owing to its anti-inflammatory properties, *C. officinalis* enhances wound healing by promoting the proliferation and migration of fibroblasts at the wound site. In vivo tests demonstrated as well the advanced efficacy of alginate-based hydrogels containing *calendula* extracts. Following a 21-day treatment period, the animals exhibited a significant reduction in the duration of epithelialization and wound constriction. Enhanced epithelialization and neovascularization, comparable to normal skin, were observed in the treated groups relative to the control group [37].

**Figure 5:**
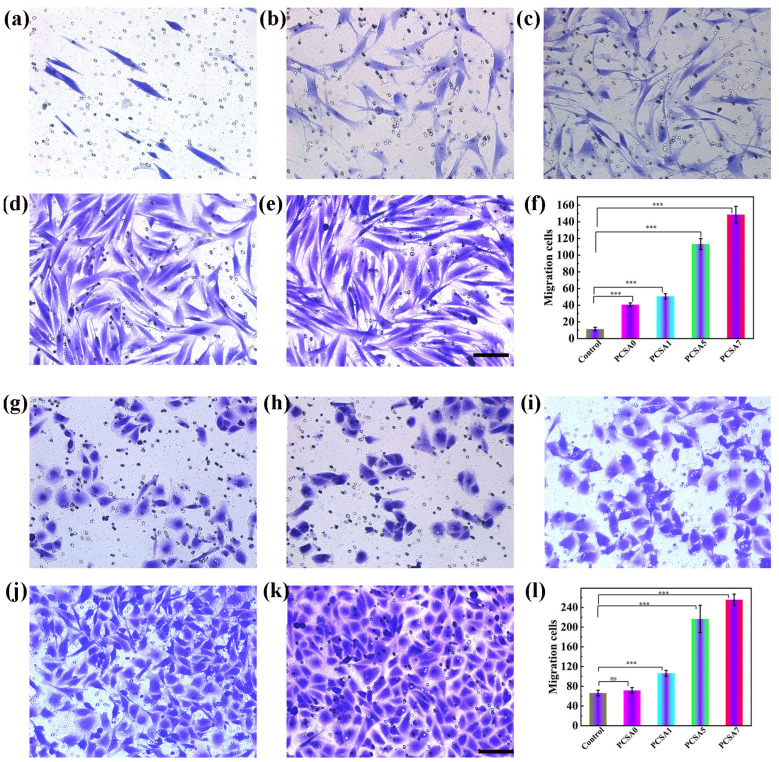
Curcumin-loaded into PVA-CS-SA hydrogels—in vitro fibroblasts and HUVECs cell migration; (**a**–**e**) Control FBs and HUVECs. Scale bar: 150 μm; (**f**) Transwell migration of FBs quantified in the histogram; (**g**–**k**) HUVECs treated with PCSA1, PCSA5, and PCSA7, respectively. Scale bar: 150 μm. (**l**) Transwell migration of HUVECs quantified in the histogram. Adapted with permission from ref. [168].

Curcumin-loaded nanoemulsion-based hydrogels demonstrated superior in vivo wound-healing efficacy and enhanced suitability for transdermal delivery. Treatment with the nanoemulsion hydrogel resulted in a greater reduction in wound diameter after 14 days compared to curcumin gel and commercial formulations. Furthermore, accelerated re-epithelialization was observed as early as day 5 of therapy [166]. In a related investigation, Bhubhanil et al. demonstrated that GG/Curcumin-AgNPs hydrogels markedly improved wound repair compared with a commercial antibacterial gel. By days 12–16, inflammatory infiltration was drastically reduced and the extent of re-epithelialization and collagen deposition increased substantially [150]. Likewise, in a full-thickness infected wound model, a 2.5% Curcumin–CS–gelatin nanoparticle hydrogel achieved almost complete closure (~93%) by day 11, while vaseline-treated controls remained largely unhealed. Immunofluorescence analysis revealed a seven-fold rise in the M2/M1 macrophage polarization ratio (7.17 ± 2.18) and significant enhancement of angiogenesis, reflected by elevated densities of α-SMA-positive arterioles (~202 mm^−2^) and vWF-positive vessels (~261 mm^−2^) [167]. Additionally, curcumin loaded into PVA-CS-SA hydrogels exhibited superior efficacy in the treatment of diabetic chronic wounds. These hydrogels significantly enhanced human skin fibroblast migration—approximately 13.5-fold within 24 h—indicating a strong stimulatory effect on cellular migration (Figure 5). Similar patterns were observed in the case of Human umbilical vein endothelial cells (HUVECs) [168].

Extracts of CA have demonstrated pronounced regenerative capacity. In vitro experiments revealed a marked rise in scratch-closure and cell-migration rates—from 73.75% to 99.56% within 24 h—and a 1.7-fold increase in type I collagen gene expression after 48 h, attributed to the triterpenoid compounds characteristic of CA [111]. Consistent in vivo results showed faster wound contraction within 10 days, together with enhanced neovascularization, increased epithelial thickness, newly formed hair follicles, and improved collagen deposition [78]. Collectively, these findings indicate that CA-enriched hydrogels effectively modulate inflammation and provide a microenvironment conducive to tissue regeneration.

Hydrogels containing chamomile (*Matricaria chamomilla* L.) extract also yielded notable outcomes, achieving approximately 85% wound closure after 14 days of treatment and promoting angiogenesis and collagen synthesis. These bioactive dressings supported complete re-epithelialization and the regeneration of structurally intact skin [75].

In a different context, grape-seed extracts (4%) were used to produce a biocompatible hydrogel composed of calcium silicate nanowires and SA for the treatment of melanoma tumors developed in Balb/c mice. By day 15, the wound had nearly healed without any signs of tumor recurrence. The grape extracts induced superior and regulated photothermal properties under NIR irradiation, hence improving the destruction of melanoma cells [169].

Advanced wound-healing performance has also been reported for HP (St John’s wort) extracts. When macerated extracts of HP were encapsulated within PLGA- and polylactic acid (PLA)-based nanoparticle–hydrogel formulations to enhance wound healing efficacy. In vivo studies using a full-thickness excisional wound model (male New Zealand rabbits) revealed significantly accelerated wound contraction, re-epithelialization, and collagen deposition in treated groups compared with controls. The formulation containing HP macerated in Nigella sativa oil showed the most pronounced healing effect. Histological analyses confirmed improved tissue regeneration without signs of irritation, underscoring the biocompatibility of the formulation. Overall, these findings demonstrate the strong therapeutic potential of nanoparticle-loaded HP hydrogels for enhanced wound repair [170]. Similarly, liposomal HP macerate incorporated into a hydrogel matrix has been shown to have superior wound healing scores on days 4, 8, and 12 of treatment. Histological analysis revealed increased epithelialization, denser collagen deposition, and comparable angiogenesis. Immunohistochemical examination indicated enhanced epithelial and vascular regeneration [41]. Furthermore, a bioactive hydrogel for wound healing was created by integrating HA callus extract into a PVA/CS/SA hydrogel. In vitro, the hydrogel exhibited superior cytocompatibility with human fibroblasts, promoting cell attachment and proliferation while suppressing microbial growth, including antibiotic-resistant strains. In vivo, by using a full-thickness murine wound model, the HP-loaded hydrogels markedly accelerated wound closure and re-epithelialization, facilitating angiogenesis and collagen deposition. Histological and immunohistochemical evaluations demonstrated significant decreases in inflammatory infiltration and fibrosis-associated markers, indicating that the formulation not only accelerates healing but also reduces excessive scar formation. These results highlight the therapeutic potential of HP-based hydrogels as advanced wound dressings that provide antibacterial protection, facilitate tissue regeneration, and inhibit fibrosis within a single biocompatible formulation [94].

**Table 3:** Non-standardized natural and bee-derived extracts in hydrogel-based wound dressings.

Natural Agents	Type of Extraction	Polymeric Composition	Therapeutic/Biological Effects
*Aloe vera*	Fresh leaves (crude gel) [171]	0.7% PVA	Enhances fibroblast proliferation; Exhibits strong wound healing potential.
	Fresh leaves [101]	3% PVA	Accelerated wound closure; Improves re-epithelialization and reduces inflammation.
	Fresh leaves [172]	2% SF/2% PVP	Enhances cellular proliferation and migration; Reduces inflammation; Promotes granulation tissue formation and accelerated re-epithelialization.
	Fresh leaves [96]	8% PVA–2% CS	Non-cytotoxic; Exhibits antibacterial activity and excellent wound dressing potential.
	Fresh leaves [173]	12% PAN–1% TG	Significantly increases fibroblast viability; Demonstrates excellent cytocompatibility
*Calendula* *officinalis* (*Marigold*)	Macerated extract [69]	7.2% AAm–0.5% MC	Promotes tissue regeneration and accelerates the healing process.
	Commercial Extract [102]	2% CMC–5%PVA	Minimizes apoptosis in human dermal fibroblasts; Exhibits antimicrobial efficacy against *S. aureus* and *E. coli.*
*Centella* *asiatica*	Macerated extract 95% EtOH [103]	8% PVA–5% PEG	Non-irritant and biocompatible; Accelerates wound contraction and epithelialization; Promotes complete wound closure with thin epidermis formation by day 5.
*Chamomile*	Ultrasonic extract (water/ethanol 3:1) [73]	1% TG	Exhibits antimicrobial activity (80% of *E. coli*, 90% of *S. aureus*, and 92% of *C. albicans*); Demonstrates anti-inflammatory and skin-protective potential.
	Fresh leaves [72]	15% AA–5% Cold/ Hot Starch	Non-cytotoxic (>70% cell viability); Promoted fibroblast proliferation and cell regeneration.
	Ethanolic extract (50%) [174]	3% CS–2% Agarose	Antibacterial activity (inhibition zones: *E. coli* 7.5 mm, *S. aureus* 12.7 mm); Supports NIH 3T3 fibroblast adhesion, proliferation (~94% viability at day 7); Bioactive flavonoids and tannins contribute to antioxidant and wound-healing activity.
	Ethanolic extract (70%) [75]	12% PAN–2% SA– 2% Gelatin	Antibacterial activity (23 ± 1 mm *S. aureus*, 12 ± 2 mm *E. coli*); Cytocompatible (>100% L929 viability); Enhances angiogenesis, collagen deposition, and wound closure (~85% after 28 days); Reduces inflammation and necrosis.
*Curcumin* (*Curcuma longa*)	Aqueous extract [175]	(1–2.5)% CS–5% PAAM	Enhances antibacterial efficiency and biocompatibility; Curcuminoids contribute to anti-inflammatory and wound-healing activity via microbial inhibition and tissue regeneration.
*Green Tea*	Fresh leaves [128]	3% CS–10% PVA	Demonstrates concentration-dependent antioxidant activity (DPPH scavenging 20–80%; ABTS scavenging 2–50%).
	Fresh leaves [82]	2% CS–10% PVA	Antibacterial activity against *E. coli* and *S. aureus*; Non-cytotoxicity (L929 viability >70%); Promotes wound closure (~98–99% by day 12); Reduces inflammation and enhanced re-epithelialization.
*Neem* (*Azadirachta* *indica*)	Fresh leaves [104]	2% CS	Exhibits antibacterial activity against *S. aureus*; Enhances wound healing efficiency; Displays anti-inflammatory effects and promotes a moist wound environment.
	Fresh leaves [25]	1.25% GG–0.75% SF	Demonstrates moderate antibacterial activity (26.5 ± 0.9 mm inhibition zone); Free of microbial contamination

### 4.2. Synergistic Effects of Natural Extracts in Hydrogel-Based Wound Dressings

Beyond the action of single bioactive components, numerous studies have explored the incorporation of multiple natural extracts into hydrogel matrices to exploit their synergistic therapeutic potential. Such combinations often result in amplified antibacterial, antioxidant, and regenerative activities, reflecting a complementary or additive interaction among phytochemical constituents and leading to improved wound-healing performance.

**Figure 6:**
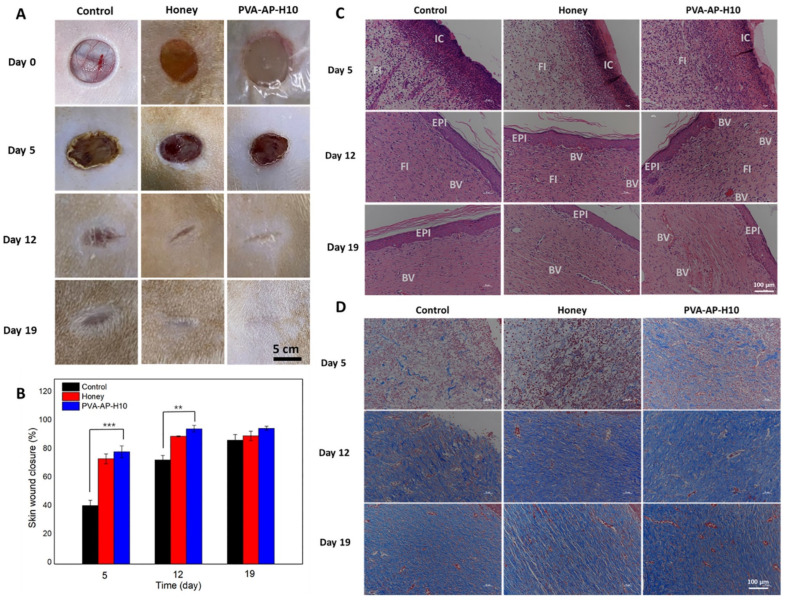
(**A**) Representative photographs illustrating the wound healing progression at selected time intervals for the control (blank), honey, and PVA-*Aloe vera*-honey hydrogel; (**B**) Percentage of wound closure evaluated over 19 days; (**C**) Histological micrographs of wound tissues collected on days 5, 12, and 19 and stained with H&E (EPI: epidermis; BV: blood vessels; FI: fibroblasts; IC: inflammatory cells); (**D**) Histopathological sections of wounds stained with Masson’s trichrome on days 5, 12, and 19 (scale bar = 100 µm). Adapted with permission from ref. [89].

Hydrogels containing both *Aloe vera* and honey exhibited excellent in vitro biocompatibility, characterized by high fibroblast viability and minimal cytotoxicity in MTT assays. The combined natural compounds effectively reduced haemolysis and markedly enhanced fibroblast adhesion and proliferation after 5 days of incubation, indicating robust cell–matrix interactions that support tissue regeneration [105]. Likewise, the integration of *Aloe vera* and honey within a PVA hydrogel network produced complementary biological effects, minimizing erythrocyte damage while promoting matrix formation and early tissue regeneration (Figure 6). The synergistic interplay between *Aloe vera* acemannan polysaccharides and honey-derived anti-inflammatory and antibacterial compounds enhanced fibroblast activity and collagen deposition, resulting in a dual therapeutic action that accelerates and optimizes wound healing [89].

An Alginate-gelatin hydrogel co-loaded with nanosilver, *Aloe vera*, curcumin, and *Calendula officinalis* extract demonstrated significant in vitro synergy, achieving approximately 98% artificial wound closure through enhanced migration and proliferation of V79 fibroblast cell [176]. Similarly, the combined incorporation of *Aloe vera* and *Curcuma longa* extracts into a gelatin–PEG–CS matrix resulted in superior in vitro and in vivo healing responses compared with single-extract or unloaded systems. The dual-extract hydrogel promoted higher dermal cell proliferation and viability, while in rat wound models, nearly complete wound closure was achieved within ten days, accompanied by rapid contraction and re-epithelialization. The combined antioxidant and anti-inflammatory properties of acemannan and curcumin stimulated fibroblast proliferation and collagen synthesis, confirming the synergistic therapeutic efficacy of these dual-extract hydrogels in accelerating tissue regeneration [133].

In a complementary study, the co-administration of *Aloe vera* extract with adipose-derived stem cells within a hydrogel framework achieved outstanding results in a rat burn model, attaining 99.4% wound closure by day 14 without visible inflammation or erythema. The treatment significantly down-regulated IL-1β while up-regulating TGF-β1 expression during the mid-healing phase, indicating balanced inflammatory modulation and tissue remodelling. Histological observations revealed accelerated re-epithelialization, abundant fibroblast proliferation, and extensive neovascularization, highlighting the synergistic interaction between *Aloe vera* bioactives and stem-cell-derived factors in promoting well-organized dermal regeneration [177]. Further evidence of synergistic enhancement was provided by hydrogel patches composed of Pluronic micelles loaded with acemannan and curcumin (2:1 ratio). The dual-loaded system exhibited improved in vivo wound contraction, stimulated early hair growth (day 10 post-injury), reduced local inflammation and granulation, and accelerated epidermal regeneration during the intermediate healing phase (days 4–8) [178].

In another formulation, curcumin-loaded, heparin-grafted PLGA nanofiber membranes (PCH NFMs) significantly increased α-SMA expression (17.4 ± 4.0%), as shown by histological and immunohistochemical analyses, indicating enhanced myofibroblast differentiation and early neovascularization. The improved healing response was attributed to the synergistic effect of curcumin’s anti-inflammatory activity and the heparin-mediated stabilization of growth factors (TGF-β1 and FGF-2), which collectively promoted angiogenesis, fibroblast activation, and accelerated tissue remodelling [179].

Additional synergistic outcomes were observed in in vitro assays of Gantrez^®^ S-97/xyloglucan hydrogel patches co-loaded with *Dioscorea bulbifera* extract and honey. At a concentration of 1 mg mL^−1^, the formulation exhibited high fibroblast viability and pronounced migratory stimulation. Scratch tests showed that both honey-loaded and unloaded hydrogels accelerated fibroblast migration within 24 h, whereas the dual-loaded Gantrez^®^ S-97/xyloglucan patches produced the most substantial enhancement after 48 h. After 72 h, the percentage of scratch-area recovery confirmed the potentiating effects of honey and *D. bulbifera* phytochemicals on fibroblast motility and proliferation [131].

Moreover, dual-loaded nanostructured lipid carrier hydrogels containing CA extract and azelaic acid, developed by Lacatusu et al., exhibited faster and more potent anti-inflammatory response than either single-component system or a commercial reference formulation. The enhanced activity was ascribed to the synergistic interplay *among calendula* flavonoids, azelaic acid, and ω-3/ω-6 fatty acids, which collectively suppressed proinflammatory cytokine release, modulated oxidative stress, and promoted tissue repair [180].

Overall, comparative evaluation of the studies summarized in this section indicates that *Aloe vera*, *Centella asiatica*, *Calendula officinalis*, and *Curcuma longa* consistently achieve the strongest regenerative responses, including faster re-epithelialization, enhanced collagen deposition, and attenuated inflammation across multiple in vitro and in vivo models. Other extracts, such as neem, chamomile, grape seed, green tea, and *Hypericum perforatum*, exhibit pronounced antimicrobial, antioxidant, or anti-inflammatory actions, though their performance is more sensitive to extraction method, standardization level, and formulation stability. Across all systems, key translational barriers remain related to batch variability, phytochemical instability, and the limited availability of robust clinical data, emphasizing the need for improved extract standardization and scalable manufacturing pathways.

## 5. Conclusions and Future Perspectives

Natural therapeutic extracts incorporated into hydrogel dressings demonstrate significant potential for improving wound healing owing to their multifunctional biological properties. *Aloe vera*, *Calendula officinalis* (Marigold), *Curcuma longa* (Turmeric), *Centella asiatica*, *Matricaria chamomilla* L. (Chamomile), *Azadirachta indica* (Neem), *Camellia sinensis* (Green tea), *Hypericum perforatum* (St. John’s Wort), honey, and bee-derived products represent the most widely studied natural ingredients capable of accelerating tissue regeneration, collagen synthesis, and re-epithelialization.

The extraction and purification of natural therapeutic extracts are critical for preserving their stability and bioactivity when incorporated into hydrogel wound dressings. Each extract has a unique chemical profile; thus, the selected extraction procedure must optimize yield while preserving biological functionality. Likewise, selecting a suitable incorporation strategy, whether direct mixing, in situ gelation, post-loading, or nanoencapsulation depends on the properties of both the bioactive compound and the hydrogel system. Standardizing these processes ensures reproducibility and consistent therapeutic performance in natural-extract-loaded hydrogels for wound healing. Comprehensive, rational design that combines optimum extraction–purification processes with appropriate incorporation techniques guarantees superior biocompatibility and improved wound-healing efficacy of natural-agent-loaded hydrogels.

The in vitro and in vivo results demonstrate that hydrogels loaded with natural therapeutic extracts markedly enhance wound healing through multifactorial pathways, surpassing the efficacy of unloaded or commercially available formulations. Among the most prominent examples, *Aloe vera*, *Centella asiatica*, *Calendula officinalis*, and *Curcuma longa* extracts significantly facilitate near-complete re-epithelialization within two to three weeks. Formulation of curcumin and *Hypericum perforatum* further demonstrated improved vascularization and reduced inflammation, leading to regenerative outcomes with minimal scarring. Similarly, Chamomile, Grape seed, and Neem extracts provided antimicrobial protection and reduced oxidative stress, essential for chronic and infected wound models. The integration of multiple natural therapeutic agents offers a powerful, synergetic approach to wound management, enabling the design of multifunctional hydrogels that simultaneously modulate inflammation, prevent infection, and accelerate tissue regeneration.

Future research should focus on the systematic optimization of extract ratios, release kinetics, and pathway correlations using biological profiling to fully exploit the synergistic healing potential of natural extracts in advanced hydrogel dressings. Consequently, the incorporation of different biopolymer compositions, innovative crosslinking strategies, and natural type extracts enables the design of next-generation hydrogels that simultaneously accelerate healing, prevent infection, and reduce fibrosis in acute and chronic wound environments. In addition to optimizing formulation parameters, future perspectives must address the requirements for large-scale production and safe clinical deployment of natural-extract-loaded hydrogel dressings. Ensuring batch-to-batch reproducibility, standardized extraction and purification protocols, and scalable hydrogel fabrication methods is essential for industrial translation. Furthermore, rigorous validation of sterility, biocompatibility, long-term stability, and controlled release behavior is imperative. Establishing robust quality control frameworks and safety assessments will facilitate the successful transition of these bioactive hydrogels from laboratory research to routine clinical wound-care applications.

## Data Availability

No new data were created or analyzed in this study.

## Abbreviations

The following abbreviations are used in this manuscript:

5-FU	5-Fluorouracil
AA	Acrylic acid
AAm	Acrylamide
AMPS	Acrylamido-2-methyl-1-propanesulfonic acid
BC	Bacterial cellulose
CA	*Centella asiatica*
CMC	Carboxymethylcellulose
COL	Collagen
CS	Chitosan
DMA	*N*,*N*-Dimethylacrylamide
DXS	Dextran sulfate
EGDMA EtOH	Ethylene glycol dimethacrylate Ethyl alcohol
GelMA	Gelatin methacryloyl
GG	Guar gum
Gly	Glycerol
GO	Graphene oxide
HA	Hyaluronic acid
HEC	Hydroxyethylcellulose
HP	*Hypericum perforatum* L.
HPMC	Hydroxypropyl methylcellulose
HUVECs	Human umbilical vein endothelial cells
MAAn	Methacrylic anhydride
MC MeOH	Methylcellulose Methyl alcohol
NIPAM	N-Isopropylacrylamide
PAAM	Polyacrylamide
PAN	Polyacrylonitrile
PCL	Poly-ε-caprolactone
PDMA	Poly(*N*,*N*-dimethylacrylamide)
PEG	Poly(ethylene glycol)
PEO	Polyethylene oxide
PG	Propylene glycol
PLA	Polylactic acid
PLGA	Poly(lactic-co-glycolic acid)
PVA	Polyvinyl alcohol
PVP	Polyvinylpyrrolidone
QCS	Quaternary aminated chitosan
SA	Sodium alginate
SF	Silk fibroin
TEA	Triethanolamine
TG	Tragacanth
TOCN	TEMPO oxidized cellulose nanofiber
XG	Xanthan gum
κ-CAR	Carrageenan

## References

[1] Cristea (Hohotă) A.-G., Lisă E.-L., Iacob (Ciobotaru) S., Dragostin I., Ștefan C.S., Fulga I., Anghel (Ștefan) A.M., Dragan M., Morariu I.D., Dragostin O.-M. (2025). Antimicrobial Smart Dressings for Combating Antibiotic Resistance in Wound Care. Pharmaceuticals.

[2] Herman A., Leska A., Wińska P., Herman A.P. (2025). Plant Extracts as Modulators of the Wound Healing Process—Preliminary Study. Int. J. Mol. Sci..

[3] Xie Y., Gao P., He F., Zhang C. (2022). Application of Alginate-Based Hydrogels in Hemostasis. Gels.

[4] Vitale S., Colanero S., Placidi M., Di Emidio G., Tatone C., Amicarelli F., D’Alessandro A.M. (2022). Phytochemistry and Biological Activity of Medicinal Plants in Wound Healing: An Overview of Current Research. Molecules.

[5] Revete A., Aparicio A., Cisterna B.A., Revete J., Luis L., Ibarra E., Segura González E.A., Molino J., Reginensi D. (2022). Advancements in the Use of Hydrogels for Regenerative Medicine: Properties and Biomedical Applications. Int. J. Biomater..

[6] Kucinska-Lipka J., Gubanska I., Lewandowska A., Terebieniec A., Przybytek A., Cieśliński H. (2019). Antibacterial Polyurethanes, Modified with Cinnamaldehyde, as Potential Materials for Fabrication of Wound Dressings. Polym. Bull..

[7] Hassanzadeh-Tabrizi S.A. (2024). Alginate Based Hemostatic Materials for Bleeding Management: A Review. Int. J. Biol. Macromol..

[8] Lopes A.I., Pintado M.M., Tavaria F.K. (2024). Plant-Based Films and Hydrogels for Wound Healing. Microorganisms.

[9] Lyggitsou G., Barda C., Anagnostou M., Douros A., Statha D., Karampasi C., Papantonaki A.I., Svoliantopoulos I., Sfiniadakis I., Vitsos A. (2024). Wound Healing Potential of Herbal Hydrogel Formulations of Cedrus Brevifolia Extracts in Mice. Gels.

[10] Gutterman Y., Chauser-Volfson E. (2000). The Distribution of the Phenolic Metabolites Barbaloin, Aloeresin and Aloenin as a Peripheral Defense Strategy in the Succulent Leaf Parts of *Aloe arborescens*. Biochem. Syst. Ecol..

[11] Hamman J.H. (2008). Composition and Applications of *Aloe vera* Leaf Gel. Molecules.

[12] Hutter J.A., Salman M., Stavinoha W.B., Satsangi N., Williams R.F., Streeper R.T., Weintraub S.T. (1996). Antiinflammatory C-Glucosyl Chromone from *Aloe barbadensis*. J. Nat. Prod..

[13] Maan A.A., Nazir A., Khan M.K.I., Ahmad T., Zia R., Murid M., Abrar M. (2018). The Therapeutic Properties and Applications of *Aloe vera*: A Review. J. Herb. Med..

[14] Liu C., Cui Y., Pi F., Cheng Y., Guo Y., Qian H. (2019). Extraction, Purification, Structural Characteristics, Biological Activities and Pharmacological Applications of Acemannan, a Polysaccharide from *Aloe vera*: A Review. Molecules.

[15] Gupta A., Singh R.L., Raghubir R. (2002). Antioxidant Status during Cutaneous Wound Healing in Immunocompromised Rats. Mol. Cell Biochem..

[16] Arafa N.M.S., Hummadi H.M.A., Badr G.M. (2025). The Potential of *Aloe vera* Gel Utilization for Skin Wound Healing in Rats Based on GC–MS and HPLC Chemical Profile. J. Basic Appl. Zool..

[17] Meza-Valle K.Z., Saucedo-Acuña R.A., Tovar-Carrillo K.L., Cuevas-González J.C., Zaragoza-Contreras E.A., Melgoza-Lozano J. (2021). Characterization and Topical Study of *Aloe vera* Hydrogel on Wound-Healing Process. Polymers.

[18] Movaffagh J., Khatib M., Fazly Bazzaz B.S., Taherzadeh Z., Hashemi M., Seyedian Moghaddam A., Tabatabaee S.A., Azizzadeh M., Jirofti N. (2022). Evaluation of Wound-Healing Efficiency of a Functional Chitosan/*Aloe vera* Hydrogel on the Improvement of Re-Epithelialization in Full Thickness Wound Model of Rat. J. Tissue Viability.

[19] Kotha R.R., Luthria D.L. (2019). Curcumin: Biological, Pharmaceutical, Nutraceutical, and Analytical Aspects. Molecules.

[20] Kant V., Gopal A., Kumar D., Pathak N.N., Ram M., Jangir B.L., Tandan S.K., Kumar D. (2015). Curcumin-Induced Angiogenesis Hastens Wound Healing in Diabetic Rats. J. Surg. Res..

[21] Yen Y., Pu C., Liu C., Chen Y., Chen Y., Liang C., Hsieh J., Huang H., Chen Y. (2018). Curcumin Accelerates Cutaneous Wound Healing via Multiple Biological Actions: The Involvement of TNF-α, MMP-9, A-SMA, and Collagen. Int. Wound J..

[22] Dai M., Zheng X., Xu X., Kong X., Li X., Guo G., Luo F., Zhao X., Wei Y., Qian Z. (2009). Chitosan-Alginate Sponge: Preparation and Application in Curcumin Delivery for Dermal Wound Healing in Rat. BioMed Res. Int..

[23] Chundran N.K., Husen I.R., Rubianti I. (2015). Effect of Neem Leaves Extract (*Azadirachta indica*) on Wound Healing. Althea Med. J..

[24] Olorunsola R.A., Oke F., Bagbe A.S., Ayeyinbo O.A. (2020). Evaluation of Wound Healing Potentials of Neem (Azadirachtaindica) Leaf Extract on Excision Wounds in Wistar Albino Rats. Niger. J. Anim. Prod..

[25] Nasrine A., Narayana S., Gulzar Ahmed M., Sultana R., Noushida N., Raunak Salian T., Almuqbil M., Almadani M.E., Alshehri A., Alghamdi A. (2023). Neem (*Azadirachta indica*) and Silk Fibroin Associated Hydrogel: Boon for Wound Healing Treatment Regimen. Saudi Pharm. J..

[26] Leung L.K., Su Y., Zhang Z., Chen Z.-Y., Huang Y., Chen R. (2001). Theaflavins in Black Tea and Catechins in Green Tea Are Equally Effective Antioxidants. J. Nutr..

[27] Reygaert W.C. (2014). The Antimicrobial Possibilities of Green Tea. Front. Microbiol..

[28] Lee S., Lee K.-W. (2007). Protective Effect of (−)-Epigallocatechin Gallate against Advanced Glycation Endproducts-Induced Injury in Neuronal Cells. Biol. Pharm. Bull..

[29] Kim H., Lee J., Kwon B.J., Lee M.H., Han D., Hyon S., Park J. (2014). Promotion of Full-Thickness Wound Healing Using Epigallocatechin-3-*O*-Gallate/Poly (Lactic-CO-GLycolic Acid) Membrane as Temporary Wound Dressing. Artif. Organs.

[30] Chandrika U.G., Prasad Kumara P.A.A.S. (2015). Chapter Four—Gotu Kola (*Centella asiatica*): Nutritional Properties and Plausible Health Benefits. Advances in Food and Nutrition Research.

[31] Ratz-lyko A., Arct J., Pytkowska K. (2016). Moisturizing and Antiinflammatory Properties of Cosmetic Formulations Containing *Centella asiatica* Extract. Indian J. Pharm. Sci..

[32] Hamid A.A., Shah Z.M., Muse R., Mohamed S. (2002). Characterisation of Antioxidative Activities of Various Extracts of *Centella asiatica* (L) Urban. Food Chem..

[33] Witkowska K., Paczkowska-Walendowska M., Garbiec E., Cielecka-Piontek J. (2024). Topical Application of *Centella asiatica* in Wound Healing: Recent Insights into Mechanisms and Clinical Efficacy. Pharmaceutics.

[34] Witkowska K., Paczkowska-Walendowska M., Plech T., Szymanowska D., Michniak-Kohn B., Cielecka-Piontek J. (2023). Chitosan-Based Hydrogels for Controlled Delivery of Asiaticoside-Rich *Centella asiatica* Extracts with Wound Healing Potential. Int. J. Mol. Sci..

[35] Witkowska K., Paczkowska-Walendowska M., Miklaszewski A., Plech T., Michniak-Kohn B., Swora-Cwynar E., Cielecka-Piontek J. (2025). Development of 3D-Printed Chitosan-Based Hydrogels Rich in *Centella asiatica* Extract for Enhanced Wound Healing Applications. J. Drug Deliv. Sci. Technol..

[36] Cruceriu D., Balacescu O., Rakosy E. (2018). *Calendula officinalis*: Potential Roles in Cancer Treatment and Palliative Care. Integr. Cancer Ther..

[37] Rathod L., Bhowmick S., Patel P., Sawant K. (2022). Calendula Flower Extract Loaded PVA Hydrogel Sheet for Wound Management: Optimization, Characterization and in-Vivo Study. J. Drug Deliv. Sci. Technol..

[38] Martins M.D., Marques M.M., Bussadori S.K., Martins M.A.T., Pavesi V.C.S., Mesquita-Ferrari R.A., Fernandes K.P.S. (2009). Comparative Analysis between *Chamomilla recutita* and Corticosteroids on Wound Healing. An In Vitro and In Vivo Study. Phytother. Res..

[39] Salehi H., Mehrasa M., Nasri-Nasrabadi B., Doostmohammadi M., Seyedebrahimi R., Davari N., Rafienia M., Hosseinabadi M.E., Agheb M., Siavash M. (2017). Effects of Nanozeolite/Starch Thermoplastic Hydrogels on Wound Healing. J. Res. Med. Sci..

[40] Yadollah-Damavandi S., Chavoshi-Nejad M., Jangholi E., Nekouyian N., Hosseini S., Seifaee A., Rafiee S., Karimi H., Ashkani-Esfahani S., Parsa Y. (2015). Topical *Hypericum perforatum* Improves Tissue Regeneration in Full-Thickness Excisional Wounds in Diabetic Rat Model. Evid. Based Complement. Altern. Med..

[41] Kurt A.A., Ibrahim B., Çınar H., Atsü A.N., Bursalıoğlu E.O., Bayır İ., Özmen Ö., Aslan İ. (2025). Nanoemulsion Hydrogel Delivery System of *Hypericum perforatum* L.: In Silico Design, In Vitro Antimicrobial–Toxicological Profiling, and In Vivo Wound-Healing Evaluation. Gels.

[42] Ahmad P., Jaleel C.A., Salem M.A., Nabi G., Sharma S. (2010). Roles of Enzymatic and Nonenzymatic Antioxidants in Plants during Abiotic Stress. Crit. Rev. Biotechnol..

[43] Mandal M.D., Mandal S. (2011). Honey: Its Medicinal Property and Antibacterial Activity. Asian Pac. J. Trop. Biomed..

[44] Abd Jalil M.A., Kasmuri A.R., Hadi H. (2017). Stingless Bee Honey, the Natural Wound Healer: A Review. Skin. Pharmacol. Physiol..

[45] Mukhopadhyay A., Rajput M., Barui A., Chatterjee S.S., Pal N.K., Chatterjee J., Mukherjee R. (2020). Dual Cross-Linked Honey Coupled 3D Antimicrobial Alginate Hydrogels for Cutaneous Wound Healing. Mater. Sci. Eng. C.

[46] El-Kased R.F., Amer R.I., Attia D., Elmazar M.M. (2017). Honey-Based Hydrogel: In Vitro and Comparative In Vivo Evaluation for Burn Wound Healing. Sci. Rep..

[47] Rajesh A., Lone S.A., Ramasubburayan R., Sikkanthar S., Thajuddin N., Lee S.-Y., Kim J.-W., MubarakAli D. (2023). A Systemic Review on *Aloe vera* Derived Natural Biomaterials for Wound Healing Applications. Biocatal. Agric. Biotechnol..

[48] Kumari A., Raina N., Wahi A., Goh K.W., Sharma P., Nagpal R., Jain A., Ming L.C., Gupta M. (2022). Wound-Healing Effects of Curcumin and Its Nanoformulations: A Comprehensive Review. Pharmaceutics.

[49] Cedillo-Cortezano M., Martinez-Cuevas L.R., López J.A.M., Barrera López I.L., Escutia-Perez S., Petricevich V.L. (2024). Use of Medicinal Plants in the Process of Wound Healing: A Literature Review. Pharmaceuticals.

[50] Mssillou I., Bakour M., Slighoua M., Laaroussi H., Saghrouchni H., Ez-Zahra Amrati F., Lyoussi B., Derwich E. (2022). Investigation on Wound Healing Effect of Mediterranean Medicinal Plants and Some Related Phenolic Compounds: A Review. J. Ethnopharmacol..

[51] Diniz L.R.L., Calado L.L., Duarte A.B.S., de Sousa D.P. (2023). *Centella asiatica* and Its Metabolite Asiatic Acid: Wound Healing Effects and Therapeutic Potential. Metabolites.

[52] Ejiohuo O., Folami S., Maigoro A.Y. (2024). Calendula in Modern Medicine: Advancements in Wound Healing and Drug Delivery Applications. Eur. J. Med. Chem. Rep..

[53] Dai Y.-L., Li Y., Wang Q., Niu F.-J., Li K.-W., Wang Y.-Y., Wang J., Zhou C.-Z., Gao L.-N. (2022). Chamomile: A Review of Its Traditional Uses, Chemical Constituents, Pharmacological Activities and Quality Control Studies. Molecules.

[54] El-Sakhawy M., Salama A., Tohamy H.-A.S. (2023). Applications of Propolis-Based Materials in Wound Healing. Arch. Dermatol. Res..

[55] Jasso de Rodríguez D., Hernández-Castillo D., Rodríguez-García R., Angulo-Sánchez J.L. (2005). Antifungal Activity In Vitro of *Aloe vera* Pulp and Liquid Fraction against Plant Pathogenic Fungi. Ind. Crops Prod..

[56] Noori A.S., Mageed N.F., Abdalameer N.K., Mohammed M.K.A., Mazhir S.N., Ali A.H., Jaber N.A., Mohammed S.H. (2022). The Histological Effect of Activated *Aloe vera* Extract by Microwave Plasma on Wound Healing. Chem. Phys. Lett..

[57] Kim S., Asnin L., Assefa A.D., Ko E.Y., Sharma K., Park S.W. (2014). Extraction of Antioxidants from *Aloe vera* Leaf Gel: A Response Surface Methodology Study. Food Anal. Methods.

[58] Maneechan W., Khumfu P., Charoensit P., Tuanchai A., Ross S., Ross G.M., Ngoenkam J., Viyoch J. (2025). Bioactive Hydrogel Scaffolds Integrating Chitosan, Silk Fibroin, and *Aloe vera* Extract for Enhanced Cartilage Tissue Regeneration. Polymers.

[59] Jales S.T.L., Barbosa R.d.M., de Albuquerque A.C., Duarte L.H.V., da Silva G.R., Meirelles L.M.A., da Silva T.M.S., Alves A.F., Viseras C., Raffin F.N. (2022). Development and Characterization of *Aloe vera* Mucilaginous-Based Hydrogels for Psoriasis Treatment. J. Compos. Sci..

[60] Bashipour F., Ghoreishi S.M. (2014). Response Surface Optimization of Supercritical CO2 Extraction of α-Tocopherol from Gel and Skin of *Aloe vera* and Almond Leaves. J. Supercrit. Fluids.

[61] Rodrigues C.L., Tomoda B.T., Viganó J., Braga A.R.C., de Moraes M.A., Veggi P.C. (2024). Production and Characterization of Silk Fibroin–*Aloe vera* Hydrogel: A Study on Extraction, Hydrogel Properties, and Release Mechanism. ACS Omega.

[62] Bashipour F., Ghoreishi S.M. (2012). Experimental Optimization of Supercritical Extraction of β-Carotene from *Aloe barbadensis* Miller via Genetic Algorithm. J. Supercrit. Fluids.

[63] Hu Q., Hu Y., Xu J. (2005). Free Radical-Scavenging Activity of *Aloe vera* (*Aloe barbadensis* Miller) Extracts by Supercritical Carbon Dioxide Extraction. Food Chem..

[64] Ivanovic J., Zizovic I., Petrovic S., Skala D. (2009). The Analysis of Different Processes of Extraction: Yield of Extracts Obtained from *Aloe vera* (*Aloe barbadensis* Miller) and Sweet Bay (*Laurus nobilis* L.) and the Exergy Analysis of Applied Processes. Chem. Ind. Chem. Eng. Q..

[65] Tanaka M., Yamada M., Toida T., Iwatsuki K. (2012). Safety Evaluation of Supercritical Carbon Dioxide Extract of *Aloe vera* Gel. J. Food Sci..

[66] Malavika J.P., Shobana C., Ragupathi M., Kumar P., Lee Y.S., Govarthanan M., Selvan R.K. (2021). A Sustainable Green Synthesis of Functionalized Biocompatible Carbon Quantum Dots from *Aloe barbadensis* Miller and Its Multifunctional Applications. Environ. Res..

[67] Dinda M., Mazumdar S., Das S., Ganguly D., Dasgupta U.B., Dutta A., Jana K., Karmakar P. (2016). The Water Fraction of *Calendula officinalis* Hydroethanol Extract Stimulates In Vitro and In Vivo Proliferation of Dermal Fibroblasts in Wound Healing. Phytother. Res..

[68] Gavan A., Colobatiu L., Hanganu D., Bogdan C., Olah N., Achim M., Mirel S. (2022). Development and Evaluation of Hydrogel Wound Dressings Loaded with Herbal Extracts. Processes.

[69] Ferreira L.M.d.M.C., Bandeira E.d.S., Gomes M.F., Lynch D.G., Bastos G.N.T., Silva-Júnior J.O.C., Ribeiro-Costa R.M. (2023). Polyacrylamide Hydrogel Containing Calendula Extract as a Wound Healing Bandage: In Vivo Test. Int. J. Mol. Sci..

[70] Possa G.d.O.K., Chopek S., Pereira A.V., Koga A.Y., Oliveira M.R.P.d., Costa M.D.M. (2024). Calendula Glycolic Extract Enhances Wound Healing of Alginate Hydrogel. Acta Cir. Bras..

[71] Nicolaus C., Junghanns S., Hartmann A., Murillo R., Ganzera M., Merfort I. (2017). In Vitro Studies to Evaluate the Wound Healing Properties of *Calendula officinalis* Extracts. J. Ethnopharmacol..

[72] Jamroży M., Głąb M., Kudłacik-Kramarczyk S., Drabczyk A., Gajda P., Tyliszczak B. (2022). The Impact of the *Matricaria chamomilla* L. Extract, Starch Solution and the Photoinitiator on Physiochemical Properties of Acrylic Hydrogels. Materials.

[73] Ghayempour S., Montazer M. (2016). A Robust Friendly Nano-Encapsulated Plant Extract in Hydrogel Tragacanth Gum on Cotton Fabric through One Single Step in-Situ Synthesis and Fabrication. Cellulose.

[74] Sellappan L.K., Sanmugam A., Manoharan S. (2021). Fabrication of Dual Layered Biocompatible Herbal Biopatch from Biological Waste for Skin—Tissue Regenerative Applications. Int. J. Biol. Macromol..

[75] Moshfeghi T., Najmoddin N., Arkan E., Hosseinzadeh L. (2024). A Multifunctional Polyacrylonitrile Fibers/Alginate-Based Hydrogel Loaded with Chamomile Extract and Silver Sulfadiazine for Full-Thickness Wound Healing. Int. J. Biol. Macromol..

[76] Azis H.A., Taher M., Ahmed A.S., Sulaiman W.M.A.W., Susanti D., Chowdhury S.R., Zakaria Z.A. (2017). In Vitro and In Vivo Wound Healing Studies of Methanolic Fraction of *Centella asiatica* Extract. South Afr. J. Bot..

[77] Kulawik-Pióro A., Osak E., Mendrycka M., Trześniewska-Ofiara Z. (2023). Bigels as Novel Systems for the Delivery Active Compounds from *Centella asiatica*. Soft Mater..

[78] Wang X., Zhang Y., Song A., Wang H., Wu Y., Chang W., Tian B., Xu J., Dai H., Ma Q. (2024). A Printable Hydrogel Loaded with Medicinal Plant Extract for Promoting Wound Healing. Adv. Healthc. Mater..

[79] Elgamily H.M., Safwat E.M., Youssef A.M. (2025). Development of a Novel Agarose/Nano-Hydroxyapatite/Grape Seed Extract Hydrogel for Biomimetic Remineralization of Demineralized Human Enamel (An In-Vitro Study). Sci. Rep..

[80] Morales C., Roeckel M., Fernández K. (2014). Microscopic Modeling of País Grape Seed Extract Absorption in the Small Intestine. AAPS PharmSciTech.

[81] Priya, Sharma A.K., Kaith B.S., Arora S., Simran, Bhagyashree (2022). Synthesis of Gelatin and Green Tea Based Stretchable Self-Healing Material of Biomedical Importance. React. Funct. Polym..

[82] Aldakheel F., Mohsen D., El Sayed M., Alawam K., Binshaya A., Alduraywish S. (2023). Silver Nanoparticles Loaded on Chitosan-g-PVA Hydrogel for the Wound-Healing Applications. Molecules.

[83] Jena A., Sahoo N.K., Sahoo P.K., Mishra S., Rout P.R., Zhong Y. (2025). Design of a Novel Green Synthesized ZVS/S-RGO -*Deinococcus radiodurans* R1 Chitosan Hydrogel Beads for Enhanced Recovery of Europium. J. Clean. Prod..

[84] Ravindra S., Murali Mohan Y., Narayana Reddy N., Mohana Raju K. (2010). Fabrication of Antibacterial Cotton Fibres Loaded with Silver Nanoparticles via “Green. Approach”. Colloids Surf. A Physicochem. Eng. Asp..

[85] Pal P., Syed S.S., Banat F. (2017). Soxhlet Extraction of Neem Pigment to Synthesize Iron Oxide Nanoparticles and Its Catalytic and Adsorption Activity for Methylene Blue Removal. Bionanoscience.

[86] Polaquini S.R.B., Svidzinski T.I.E., Kemmelmeier C., Gasparetto A. (2006). Effect of Aqueous Extract from Neem (*Azadirachta indica* A. Juss) on Hydrophobicity, Biofilm Formation and Adhesion in Composite Resin by Candida Albicans. Arch. Oral. Biol..

[87] Seriana I., Akmal M., Darusman D., Wahyuni S., Khairan K., Sugito S. (2021). Neem Leaf (*Azadirachta indica* A. Juss) Ethanolic Extract on the Liver and Kidney Function of Rats. Sci. World J..

[88] Nowak A., Muzykiewicz-Szymańska A., Perużyńska M., Kucharska E., Kucharski Ł., Jakubczyk K., Niedźwiedzka-Rystwej P., Stefanowicz-Hajduk J., Droździk M., Majtan J. (2025). Assessment of In Vitro Skin Permeation and Accumulation of Phenolic Acids from Honey and Honey-Based Pharmaceutical Formulations. BMC Complement. Med. Ther..

[89] Zhang Q., Zhang M., Wang T., Chen X., Li Q., Zhao X. (2022). Preparation of Aloe Polysaccharide/Honey/PVA Composite Hydrogel: Antibacterial Activity and Promoting Wound Healing. Int. J. Biol. Macromol..

[90] Jansen-Alves C., Maia D.S.V., Krumreich F.D., Crizel-Cardoso M.M., Fioravante J.B., da Silva W.P., Borges C.D., Zambiazi R.C. (2019). Propolis Microparticles Produced with Pea Protein: Characterization and Evaluation of Antioxidant and Antimicrobial Activities. Food Hydrocoll..

[91] Amorim J.D.P., Nascimento H.A., Silva Junior C.J.G., Medeiros A.D.M., Silva I.D.L., Costa A.F.S., Vinhas G.M., Sarubbo L.A. (2022). Obtainment of Bacterial Cellulose with Added Propolis Extract for Cosmetic Applications. Polym. Eng. Sci..

[92] Ferreira L.M.d.M.C., Cruz N.F.d., Lynch D.G., Costa P.F.d., Salgado C.G., Silva-Júnior J.O.C., Rossi A., Ribeiro-Costa R.M. (2024). Hydrogel Containing Propolis: Physical Characterization and Evaluation of Biological Activities for Potential Use in the Treatment of Skin Lesions. Pharmaceuticals.

[93] Jarzębski M., Smułek W., Baranowska H.M., Masewicz Ł., Kobus-Cisowska J., Ligaj M., Kaczorek E. (2020). Characterization of St. John’s Wort (*Hypericum perforatum* L.) and the Impact of Filtration Process on Bioactive Extracts Incorporated into Carbohydrate-Based Hydrogels. Food Hydrocoll..

[94] Zivari-Ghader T., Shokouhi B., Kosari-Nasab M., Davaran S., Hamishehkar H., Farahpour M.R., Rashidi M., Mehrali M. (2024). *Hypericum perforatum* Callus Extract-Loaded Composite Hydrogel with Diverse Bioactivities for Enhanced Wound Healing and Fibrosis Prevention. Small.

[95] Zivari-Ghader T., Hamishehkar H., Shokouhi B., Kosari-Nasab M., Farahpour M.R., Memar M.Y., Davaran S., Hanaee J., Rashidi M.-R., Mehrali M. (2024). Chitosan-Alginate Hydrogel Enriched with *Hypericum perforatum* Callus Extract for Improved Wound Healing and Scar Inhibition. ACS Appl. Mater. Interfaces.

[96] Alvandi H., Rajati H., Naseriyeh T., Rahmatabadi S.S., Hosseinzadeh L., Arkan E. (2024). Incorporation of *Aloe vera* and Green Synthesized ZnO Nanoparticles into the Chitosan/PVA Nanocomposite Hydrogel for Wound Dressing Application. Polym. Bull..

[97] Baniasadi H. (2025). State-of-the-Art in Natural Hydrogel-Based Wound Dressings: Design, Functionalization, and Fabrication Approaches. Adv. Colloid. Interface Sci..

[98] Luo Z., Wang Y., Xu Y., Wang J., Yu Y. (2023). Modification and Crosslinking Strategies for Hyaluronic Acid-based Hydrogel Biomaterials. Smart Med..

[99] Luo Z., Wang Y., Li J., Wang J., Yu Y., Zhao Y. (2023). Tailoring Hyaluronic Acid Hydrogels for Biomedical Applications. Adv. Funct. Mater..

[100] Hanif W., Hardiansyah A., Randy A., Asri L.A.T.W. (2021). Physically Crosslinked PVA/Graphene-Based Materials/*Aloe vera* Hydrogel with Antibacterial Activity. RSC Adv..

[101] Kenawy E.-R., El-Meligy M.A., Ghaly Z.S., Kenawy M.E., Kamoun E.A. (2024). Novel Physically-Crosslinked Caffeine and Vitamin C-Loaded PVA/*Aloe vera* Hydrogel Membranes for Topical Wound Healing: Synthesis, Characterization and In-Vivo Wound Healing Tests. J. Polym. Environ..

[102] Huang W.-H., Hung C.-Y., Chiang P.-C., Lee H., Lin I.-T., Lai P.-C., Chan Y.-H., Feng S.-W. (2023). Physicochemical Characterization, Biocompatibility, and Antibacterial Properties of CMC/PVA/*Calendula officinalis* Films for Biomedical Applications. Polymers.

[103] Ahmed A.S., Taher M., Mandal U.K., Jaffri J.M., Susanti D., Mahmood S., Zakaria Z.A. (2019). Pharmacological Properties of *Centella asiatica* Hydrogel in Accelerating Wound Healing in Rabbits. BMC Complement. Altern. Med..

[104] Iqbal Y., Raouf Malik A., Iqbal T., Hammad Aziz M., Ahmed F., Abolaban F.A., Mansoor Ali S., Ullah H. (2021). Green Synthesis of ZnO and Ag-Doped ZnO Nanoparticles Using *Azadirachta indica* Leaves: Characterization and Their Potential Antibacterial, Antidiabetic, and Wound-Healing Activities. Mater. Lett..

[105] Saberian M., Seyedjafari E., Zargar S.J., Mahdavi F.S., Sanaei-rad P. (2021). Fabrication and Characterization of Alginate/Chitosan Hydrogel Combined with Honey and *Aloe vera* for Wound Dressing Applications. J. Appl. Polym. Sci..

[106] Sabouri Moghadam A., Mirmohammad Meiguni M.S., Salami M., Askari G., Emam-Djomeh Z., Miran M., Buttar H.S., Brennan C. (2024). Characterization of Physicochemical Properties of Mung Bean Protein Isolate and κ-Carrageenan Hydrogel as a Delivery System for Propolis Extract. Food Res. Int..

[107] Lu P., Ruan D., Huang M., Tian M., Zhu K., Gan Z., Xiao Z. (2024). Harnessing the Potential of Hydrogels for Advanced Therapeutic Applications: Current Achievements and Future Directions. Signal Transduct. Target. Ther..

[108] Shanmugha Mary A., Mani A., Ghosh S., Rajaram K. (2024). Phage Embedded Gelatin, Alginate and Gelatin/Alginate-Starch Based Hydrogels for Topical Bactericidal Applications against Multi-Drug Resistant *Pseudomonas aeruginosa*. J. Indian Chem. Soc..

[109] Wang X., Yang J., Zhao Q., Xie X., Deng F., Wang Z., Jiang K., Li X., Liu H., Shi Z. (2024). A Tissue-Adhesive, Mechanically Enhanced, Natural *Aloe vera*-Based Injectable Hydrogel for Wound Healing: Macrophage Mediation and Collagen Proliferation. Int. J. Biol. Macromol..

[110] Dey A., Bera R., Chakrabarty D. (2015). Influence of *Aloe vera* on the Properties of N-Vinylpyrrolidone-Acrylamide Copolymer Hydrogel. Mater. Chem. Phys..

[111] Rashedi S., Heydari P., Kharazi A.Z., Varshosaz J., Sheikholeslam M. (2025). Chitosan/Poly (Β-amino Ester) Hydrogel by Controlled Release of *Centella asiatica* Promoted Wound Healing through Improved Collagen Expression and Antibacterial and Anti-inflammatory Properties. Polym. Eng. Sci..

[112] Sampath Udeni Gunathilake T.M., Ching Y.C., Chuah C.H., Illias H.A., Ching K.Y., Singh R., Nai-Shang L. (2018). Influence of a Nonionic Surfactant on Curcumin Delivery of Nanocellulose Reinforced Chitosan Hydrogel. Int. J. Biol. Macromol..

[113] Alves C., Ribeiro A., Pinto E., Santos J., Soares G. (2022). Exploring Z-Tyr-Phe-OH-Based Hydrogels Loaded with Curcumin for the Development of Dressings for Wound Healing. J. Drug Deliv. Sci. Technol..

[114] Bai Q., Hu F., Gou S., Gao Q., Wang S., Zhang W., Zhang Y., Lu T. (2024). Curcumin-Loaded Chitosan-Based Hydrogels Accelerating *S. Aureus*-Infected Wound Healing. Int. J. Biol. Macromol..

[115] Gupta S., Ghoshal G. (2024). Plant Protein Hydrogel as a Delivery System of Curcumin: Characterization and In Vitro Release Kinetics. Food Bioprod. Process..

[116] Jayaramudu T., Raghavendra G.M., Varaprasad K., Raju K.M., Sadiku E.R., Kim J. (2016). 5-Fluorouracil Encapsulated Magnetic Nanohydrogels for Drug-delivery Applications. J. Appl. Polym. Sci..

[117] Jayaramudu T., Varaprasad K., Sadiku E.R., Kim H.C., Kim J. (2018). Preparation of Antibacterial Temperature-sensitive Silver-nanocomposite Hydrogels from *N.* -isopropylacrylamide with Green Tea. J. Appl. Polym. Sci..

[118] Abid Mustafa M., Rashid Hussain H., Akbar Khan J., Ahmad N., Bashir S., Asad M., Saeed Shah H., Ali Khan A., Malik A., Fatima S. (2025). Development and In Vitro Characterization of *Azadirachta indica* Gum Grafted Polyacrylamide Based PH-Sensitive Hydrogels to Improve the Bioavailability of Lansoprazole. Chem. Biodivers..

[119] Vigata M., Meinert C., Hutmacher D.W., Bock N. (2020). Hydrogels as Drug Delivery Systems: A Review of Current Characterization and Evaluation Techniques. Pharmaceutics.

[120] Ghasemi A.H., Farazin A., Mohammadimehr M., Naeimi H. (2022). Fabrication and Characterization of Biopolymers with Antibacterial Nanoparticles and *Calendula officinalis* Flower Extract as an Active Ingredient for Modern Hydrogel Wound Dressings. Mater. Today Commun..

[121] Pelin I.M., Silion M., Popescu I., Rîmbu C.M., Fundueanu G., Constantin M. (2023). Pullulan/Poly(Vinyl Alcohol) Hydrogels Loaded with *Calendula officinalis* Extract: Design and In Vitro Evaluation for Wound Healing Applications. Pharmaceutics.

[122] Choudhary V., Malik A. (2025). Enhanced Wound-Healing by Hydrogel from Okra Mucilage Grafted with Poly-2-Acrylamido-2-Methylpropane Sulfonic Acid (AMPS), a Stimuli-Responsive Polymer. Eur. Polym. J..

[123] Hong G.S., Choi J.Y., Suh J.S., Lim J.O., Choi J.H. (2020). Development of a Natural Matrix Hybrid Hydrogel Patch and Evaluation of Its Efficacy against Atopic Dermatitis. Appl. Sci..

[124] Hezari S., Olad A., Dilmaghani A. (2024). Investigation of Antibacterial Properties and Sustained Release of *Centella asiatica* Extract from Fe-MOF-Reinforced Gelatin-Based Hydrogels. Polym. Bull..

[125] Hezari S., Olad A., Dilmaghani A. (2025). Development of Gelatin and Modified Gelatin Hydrogels Incorporated with Aluminum-Based Metal–Organic Frameworks as a Potential Wound Dressing. Polym. Bull..

[126] Khamrai M., Banerjee S.L., Paul S., Samanta S., Kundu P.P. (2019). Curcumin Entrapped Gelatin/Ionically Modified Bacterial Cellulose Based Self-Healable Hydrogel Film: An Eco-Friendly Sustainable Synthesis Method of Wound Healing Patch. Int. J. Biol. Macromol..

[127] Luo C., Wei N., Sun X., Luo F. (2020). Fabrication of Self-healable, Conductive, and Ultra-strong Hydrogel from Polyvinyl Alcohol and Grape Seed–Extracted Polymer. J. Appl. Polym. Sci..

[128] Chuysinuan P., Chunshom N., Kotcharat P., Thanyacharoen T., Techasakul S., Ummartyotin S. (2021). The Encapsulation of Green Tea Extract in Cyclodextrin and Loading into Chitosan-Based Composites: Controlled-Release Behavior and Antioxidant Properties. J. Polym. Environ..

[129] Aminu N., Alfred-Ugbenbo D., Moradeke O., Audu Mumuni M., Muhammad Umar N., Tanko N., Raghavulu Bitra V., Tshepo Moshapa F., Monkgogi T., Siok-Yee C. (2025). Nanogel Drug Delivery System Loaded with *Azadirachta indica* A. Juss. (Neem) for Potential Treatment of Wound Infection: Development and Characterization. Beni Suef Univ. J. Basic. Appl. Sci..

[130] Sharaf S., El-Naggar M.E. (2019). Wound Dressing Properties of Cationized Cotton Fabric Treated with Carrageenan/Cyclodextrin Hydrogel Loaded with Honey Bee Propolis Extract. Int. J. Biol. Macromol..

[131] Eakwaropas P., Ngawhirunpat T., Rojanarata T., Patrojanasophon P., Opanasopit P., Nuntharatanapong N. (2022). Formulation and Optimal Design of *Dioscorea bulbifera* and Honey-Loaded Gantrez^®^/Xyloglucan Hydrogel as Wound Healing Patches. Pharmaceutics.

[132] Yang S., Wang F., Han H., Santos H.A., Zhang Y., Zhang H., Wei J., Cai Z. (2023). Fabricated Technology of Biomedical Micro-Nano Hydrogel. Biomed. Technol..

[133] Julie B.-A., John R., Natalia M.-C. (2025). Microstructural, Bioactive, and Wound-Healing Properties of Chitosan-Based Dressings with Encapsulated *Aloe vera* and *Curcuma longa* Extracts. Mater. Today Commun..

[134] Ameli S., Nourani M., Bakhshi N., Salemi B., Assadpour E., Jafari S.M. (2025). Alginate-Gelatin Composite Hydrogels for Encapsulating *Aloe vera* Extract; Optimization, Characterization, and Release Kinetics. Carbohydr. Polym. Technol. Appl..

[135] Shefa A.A., Sultana T., Park M.K., Lee S.Y., Gwon J.-G., Lee B.-T. (2020). Curcumin Incorporation into an Oxidized Cellulose Nanofiber-Polyvinyl Alcohol Hydrogel System Promotes Wound Healing. Mater. Des..

[136] Cardoso-Daodu I.M., Ilomuanya M.O., Azubuike C.P. (2022). Development of Curcumin-Loaded Liposomes in Lysine–Collagen Hydrogel for Surgical Wound Healing. Beni Suef Univ. J. Basic. Appl. Sci..

[137] Kour P., Afzal S., Gani A., Zargar M.I., Nabi Tak U., Rashid S., Dar A.A. (2022). Effect of Nanoemulsion-Loaded Hybrid Biopolymeric Hydrogel Beads on the Release Kinetics, Antioxidant Potential and Antibacterial Activity of Encapsulated Curcumin. Food Chem..

[138] Mahdian M., Akbari Asrari S., Ahmadi M., Madrakian T., Rezvani Jalal N., Afkhami A., Moradi M., Gholami L. (2023). Dual Stimuli-Responsive Gelatin-Based Hydrogel for PH and Temperature-Sensitive Delivery of Curcumin Anticancer Drug. J. Drug Deliv. Sci. Technol..

[139] Preda P., Enciu A.-M., Adiaconita B., Mihalache I., Craciun G., Boldeiu A., Aricov L., Romanitan C., Stan D., Marculescu C. (2022). New Amorphous Hydrogels with Proliferative Properties as Potential Tools in Wound Healing. Gels.

[140] Garcia-Orue I., Santos-Vizcaino E., Uranga J., de la Caba K., Guerrero P., Igartua M., Hernandez R.M. (2023). Agar/Gelatin Hydro-Film Containing EGF and *Aloe vera* for Effective Wound Healing. J. Mater. Chem. B.

[141] Darwish M.M., Elneklawi M.S., Mohamad E.A. (2023). *Aloe vera* Coated Dextran Sulfate/Chitosan Nanoparticles (*Aloe vera* @ DS/CS) Encapsulating Eucalyptus Essential Oil with Antibacterial Potent Property. J. Biomater. Sci. Polym. Ed..

[142] Karimi E., Gharib B., Rostami N., Navidpour L., Afshar M. (2019). Clinical Efficacy of a Topical Polyherbal Formulation in the Management of Fluorouracil-Associated Hand-Foot Syndrome. J. Herb. Med..

[143] Colobatiu L., Gavan A., Mocan A., Bogdan C., Mirel S., Tomuta I. (2019). Development of Bioactive Compounds-Loaded Chitosan Films by Using a QbD Approach—A Novel and Potential Wound Dressing Material. React. Funct. Polym..

[144] Chanaj-Kaczmarek J., Paczkowska M., Osmałek T., Kaproń B., Plech T., Szymanowska D., Karaźniewicz-Łada M., Kobus-Cisowska J., Cielecka-Piontek J. (2020). Hydrogel Delivery System Containing Calendulae Flos Lyophilized Extract with Chitosan as a Supporting Strategy for Wound Healing Applications. Pharmaceutics.

[145] Saha I., Roy S., Das D., Das S., Karmakar P. (2023). Topical Effect of Polyherbal Flowers Extract on Xanthan Gum Hydrogel Patch—Induced Wound Healing Activity in Human Cell Lines and Male BALB/c Mice. Biomed. Mater..

[146] Mathew D., Thomas B., Soumya P.T., Sudheep N.M. (2025). Development of Chitosan Based Hydrogels with Marigold Flower Extract: An Innovative, Low Cost, Biodegradable and Antimicrobial Solution for Enhanced Wound Healing Applications. Results Surf. Interfaces.

[147] Kuo C.-W., Chiu Y.-F., Wu M.-H., Li M.-H., Wu C.-N., Chen W.-S., Huang C.-H. (2021). Gelatin/Chitosan Bilayer Patches Loaded with Cortex *Phellodendron amurense*/*Centella asiatica* Extracts for Anti-Acne Application. Polymers.

[148] Tohidi A., Montazer M., Mianehro A., Rad M.M. (2024). Biocompatible Polysaccharide-Based Wound Dressing Comprising Cellulose Fabric Treated with Gum Tragacanth, Alginate, Bacterial Cellulose, and Chamomile Extracts. Starch-Stärke.

[149] Zakerikhoob M., Abbasi S., Yousefi G., Mokhtari M., Noorbakhsh M.S. (2021). Curcumin-Incorporated Crosslinked Sodium Alginate-g-Poly (N-Isopropyl Acrylamide) Thermo-Responsive Hydrogel as an in-Situ Forming Injectable Dressing for Wound Healing: In Vitro Characterization and In Vivo Evaluation. Carbohydr. Polym..

[150] Bhubhanil S., Talodthaisong C., Khongkow M., Namdee K., Wongchitrat P., Yingmema W., Hutchison J.A., Lapmanee S., Kulchat S. (2021). Enhanced Wound Healing Properties of Guar Gum/Curcumin-Stabilized Silver Nanoparticle Hydrogels. Sci. Rep..

[151] Chopra H., Bibi S., Mohanta Y.K., Kumar Mohanta T., Kumar S., Singh I., Saad Khan M., Ranjan Rauta P., Alshammari A., Alharbi M. (2023). In Vitro and In Silico Characterization of Curcumin-Loaded Chitosan–PVA Hydrogels: Antimicrobial and Potential Wound Healing Activity. Gels.

[152] Khodaei A., Johari N., Jahanmard F., Cecotto L., Khosravimelal S., Madaah Hosseini H.R., Bagheri R., Samadikuchaksaraei A., Amin Yavari S. (2024). Particulate 3D Hydrogels of Silk Fibroin-Pluronic to Deliver Curcumin for Infection-Free Wound Healing. Biomimetics.

[153] Yang J., Duan A., Shen L., Liu Q., Wang F., Liu Y. (2024). Preparation and Application of Curcumin Loaded with Citric Acid Crosslinked Chitosan-Gelatin Hydrogels. Int. J. Biol. Macromol..

[154] Cai J., Zhong H., Tang W., Wen F., Lv Y., Huang X., Luo J., Li P. (2024). Multiple Response Behaviors of Curcumin-Loaded Ammonium Alginate/Polyvinyl Alcohol Hydrogel and Its Application. Biomass Convers. Biorefin.

[155] Kannan P.R., Kumar C.S., Zhao R., Iqbal M.Z., Li Y., Kong X. (2025). Dual-Functional Hydrogel with Curcumin-Loaded GelMA and Silk Fibroin for Wound Healing: Characterization and In Vitro Evaluation. Mater. Today Commun..

[156] Zhang Y., Sun J., Liu Y., Sun S., Wang K. (2025). Multi-Functional Dressing with Curcumin Displays Anti-Inflammatory, Antioxidant, Angiogenic, and Collagen Regeneration Effects in Diabetic Wound Healing. J. Mater. Sci..

[157] Teixeira L.S., Sousa M., Massano F., Borges A. (2024). Exploring Grape Pomace Extracts for the Formulation of New Bioactive Multifunctional Chitosan/Alginate-Based Hydrogels for Wound Healing Applications. Food Biosci..

[158] Das P., Chakravarty T., Roy A.J., Manna S., Nandi S.K., Basak P. (2023). Sustainable Development of Draksha- Beeja Extract Loaded Gelatin and Starch-Based Green and Biodegradable Mats for Potential Tissue Engineering Applications. Sustain. Chem. Pharm..

[159] Wang T., Zhang F., Zhao R., Wang C., Hu K., Sun Y., Politis C., Shavandi A., Nie L. (2020). Polyvinyl Alcohol/Sodium Alginate Hydrogels Incorporated with Silver Nanoclusters via Green Tea Extract for Antibacterial Applications. Des. Monomers Polym..

[160] Rungrod A., Makarasen A., Patnin S., Techasakul S., Somsunan R. (2025). Design and Development of a Sprayable Hydrogel Based on Thermo/PH Dual-Responsive Polymer Incorporating *Azadirachta indica* (Neem) Extract for Wound Dressing Applications. Polymers.

[161] Abd El-Malek F.F., Yousef A.S., El-Assar S.A. (2017). Hydrogel Film Loaded with New Formula from Manuka Honey for Treatment of Chronic Wound Infections. J. Glob. Antimicrob. Resist..

[162] Kim J., Lee C.-M. (2018). Transdermal Hydrogel Composed of Polyacrylic Acid Containing Propolis for Wound Healing in a Rat Model. Macromol. Res..

[163] Saleh S., Salama A., Ali A.M., Saleh A.K., Elhady B.A., Tolba E. (2023). Egyptian Propolis Extract for Functionalization of Cellulose Nanofiber/Poly(Vinyl Alcohol) Porous Hydrogel along with Characterization and Biological Applications. Sci. Rep..

[164] Cao-Luu N.-H., Nguyen T.-V., Luong H.-V.-T., Dang H.-G., Pham H.-G. (2025). Engineered Polyvinyl Alcohol/Chitosan/Carrageenan Nanofibrous Membrane Loaded with *Aloe vera* for Accelerating Third-Degree Burn Wound Healing. Int. J. Biol. Macromol..

[165] Choudhary V., Sharma S., Malik A., Shrivastav A., Shukla P.K. (2025). In Vivo Study of Modified Okra Mucilage/Acrylic Acid Hydrogels Loaded with Ethanolic Extracts of *Calendula officinalis* for Wound Healing Application. Adv. Pharmacol. Pharm..

[166] Le T.T.N., Nguyen T.K.N., Nguyen V.M., Dao T.C.M., Nguyen H.B.C., Dang C.T., Le T.B.C., Nguyen T.K.L., Nguyen P.T.T., Dang L.H.N. (2023). Development and Characterization of a Hydrogel Containing Curcumin-Loaded Nanoemulsion for Enhanced In Vitro Antibacteria and In Vivo Wound Healing. Molecules.

[167] Cai X., He Y., Cai L., Zhan J., Li Q., Zhong S., Hou H., Wang W., Qiu X. (2023). An Injectable Elastic Hydrogel Crosslinked with Curcumin–Gelatin Nanoparticles as a Multifunctional Dressing for the Rapid Repair of Bacterially Infected Wounds. Biomater. Sci..

[168] Fan X., Huang J., Zhang W., Su Z., Li J., Wu Z., Zhang P. (2024). A Multifunctional, Tough, Stretchable, and Transparent Curcumin Hydrogel with Potent Antimicrobial, Antioxidative, Anti-Inflammatory, and Angiogenesis Capabilities for Diabetic Wound Healing. ACS Appl. Mater. Interfaces.

[169] Ma H., Zhou Q., Chang J., Wu C. (2019). Grape Seed-Inspired Smart Hydrogel Scaffolds for Melanoma Therapy and Wound Healing. ACS Nano.

[170] Kurt A.A., Ibrahim B., Cinar H., Ozmen O. (2025). Enhanced Therapeutic Potential of *Hypericum perforatum* L.: A Comprehensive In Vitro and In Vivo Evaluation of Nanoparticle-Hydrogel Formulations. J. Nanotechnol..

[171] Hajian M., Mahmoodi M., Imani R. (2017). In Vitro Assessment of Poly (Vinyl Alcohol) Film Incorporating *Aloe vera* for Potential Application as a Wound Dressing. J. Macromol. Sci. Part B.

[172] Bhar B., Chakraborty B., Nandi S.K., Mandal B.B. (2022). Silk-Based Phyto-Hydrogel Formulation Expedites Key Events of Wound Healing in Full-Thickness Skin Defect Model. Int. J. Biol. Macromol..

[173] Alvandi H., Jaymand M., Eskandari M., Aghaz F., Hosseinzadeh L., Heydari M., Arkan E. (2023). A Sandwich Electrospun Nanofibers/Tragacanth Hydrogel Composite Containing *Aloe vera* Extract and Silver Sulfadiazine as a Wound Dressing. Polym. Bull..

[174] Manoharan S., Balakrishnan P., Sellappan L.K. (2024). Fabrication of Highly Flexible Biopolymeric Chitosan/Agarose Based Bioscaffold with *Matricaria recutita* Herbal Extract for Antimicrobial Wound Dressing Applications. Int. J. Biol. Macromol..

[175] Ferfera-Harrar H., Berdous D., Benhalima T. (2018). Hydrogel Nanocomposites Based on Chitosan-g-Polyacrylamide and Silver Nanoparticles Synthesized Using *Curcuma longa* for Antibacterial Applications. Polym. Bull..

[176] Jessy Mercy D., Thirumalai A., Udayakumar S., Deepika B., Janani G., Girigoswami A., Girigoswami K. (2024). Enhancing Wound Healing with Nanohydrogel-Entrapped Plant Extracts and Nanosilver: An In Vitro Investigation. Molecules.

[177] Oryan A., Alemzadeh E., Mohammadi A.A., Moshiri A. (2019). Healing Potential of Injectable *Aloe vera* Hydrogel Loaded by Adipose-Derived Stem Cell in Skin Tissue-Engineering in a Rat Burn Wound Model. Cell Tissue Res..

[178] Sharma A., Mittal P., Yadav A., Mishra A.K., Hazari P.P., Sharma R.K. (2022). Sustained Activity of Stimuli-Responsive Curcumin and Acemannan Based Hydrogel Patches in Wound Healing. ACS Appl. Bio Mater..

[179] Liao H.T., Lai Y.-T., Kuo C.-Y., Chen J.-P. (2021). A Bioactive Multi-Functional Heparin-Grafted Aligned Poly(Lactide-Co-Glycolide)/Curcumin Nanofiber Membrane to Accelerate Diabetic Wound Healing. Mater. Sci. Eng. C.

[180] Lacatusu I., Badea G., Popescu M., Bordei N., Istrati D., Moldovan L., Seciu A.M., Panteli M.I., Rasit I., Badea N. (2017). Marigold Extract, Azelaic Acid and Black Caraway Oil into Lipid Nanocarriers Provides a Strong Anti-Inflammatory Effect In Vivo. Ind. Crops Prod..

